# A complex genomic architecture underlies reproductive isolation in a North American oriole hybrid zone

**DOI:** 10.1038/s42003-023-04532-8

**Published:** 2023-02-07

**Authors:** Jennifer Walsh, Shawn M. Billerman, Bronwyn G. Butcher, Vanya G. Rohwer, David P. L. Toews, Vicens Vila-Coury, Irby J. Lovette

**Affiliations:** 1 Fuller Evolutionary Biology Program, Cornell Lab of Ornithology, Cornell University, Ithaca, NY USA; 2 Department of Biology, Penn State University, University Park, Pennsylvania, USA; 3 Department of Ecology and Evolutionary Biology, Cornell University, Ithaca, NY USA

**Keywords:** Speciation, Genetics

## Abstract

Natural hybrid zones provide powerful opportunities for identifying the mechanisms that facilitate and inhibit speciation. Documenting the extent of genomic admixture allows us to discern the architecture of reproductive isolation through the identification of isolating barriers. This approach is particularly powerful for characterizing the accumulation of isolating barriers in systems exhibiting varying levels of genomic divergence. Here, we use a hybrid zone between two species—the Baltimore (*Icterus galbula*) and Bullock’s (*I. bullockii*) orioles—to investigate this architecture of reproductive isolation. We combine whole genome re-sequencing with data from an additional 313 individuals amplityped at ancestry-informative markers to characterize fine-scale patterns of admixture, and to quantify links between genes and the plumage traits. On a genome-wide scale, we document several putative barriers to reproduction, including elevated peaks of divergence above a generally high genomic baseline, a large putative inversion on the Z chromosome, and complex interactions between melanogenesis-pathway candidate genes. Concordant and coincident clines for these different genomic regions further suggest the coupling of pre- and post-mating barriers. Our findings of complex and coupled interactions between pre- and post-mating barriers suggest a relatively rapid accumulation of barriers between these species, and they demonstrate the complexities of the speciation process.

## Abstract (web summary)

Genome-wide analyses of admixture between two closely related and sympatric North American oriole species reveals longstanding barriers enforcing reproductive isolation.

## Introduction

Hybrid zones can be used as natural experiments to discover the processes that both facilitate and inhibit speciation. Mounting empirical and theoretical work has shown that hybridization between related species is relatively common in nature, occurring in approximately 25% of flowering plants and approximately 10% of animals^1,2^. The relevance of hybrid zones for studying the origin and maintenance of reproductive isolation has been demonstrated through many studies^3–5^, but their utility for elucidating the coupling or buildup of barrier effects to hybridization is a more recent research focus^6–8^. Research has increasingly shown that the maintenance of species boundaries often relies on the combinatorial interaction of several mechanisms^9–11^. This aggregate evolution of multiple reproductive barriers is important in preventing gene exchange, even in recently diverged species^8^. Accordingly, we can use hybrid zones to explore the idea that the process of speciation hinges not only on the evolution of individual barriers to reproduction between closely related species, but sometimes equally so on the complex pleiotropic interactions that couple these barrier effects together^8^.

Growing access to genomic data from many taxonomic groups has informed the contributions of ongoing hybridization to the evolution and maintenance of biodiversity^12–15^. With existing bioinformatic tools and increased genomic resolution, it is clear that hybridization has played a substantial role in shaping the evolutionary histories of many closely related species^16–18^. In recently diverged birds, there has been a wave of literature linking simple pre-mating signals (i.e., plumage variants) with reproductive isolation, as indicated by selection for a few divergent regions underlying pigmentation genes across an otherwise largely neutral background of low differentiation^12,19–21^. While these studies have been extremely informative in shaping our understanding of the genomics of reproductive isolation near the onset of the speciation process, there are notably fewer genomic studies that look at the accumulation of barriers in avian hybrid zones with greater levels of genomic differentiation and that are further along the speciation continuum (but see refs. ^22,23^). For example, we know that at intermediate levels of divergence, an accumulation of incompatibilities may influence hybrid fitness (through post-mating barriers), but the number of barriers may be insufficient to prevent introgression of some genomic regions^23^. While these semipermeable barriers to gene flow are important in shaping hybridization outcomes, our understanding of how pre- and post-mating barriers accumulate across the genomic landscape, and for how long following species divergence these regions can be exchanged, remains incomplete.

Here we leverage a historically prominent hybrid zone between Baltimore (*Icterus galbula*) and Bullock’s (*I. bullockii*) orioles to investigate the genomic architecture of reproductive isolation between two divergent but actively hybridizing avian species. Although they are not sister species, Baltimore and Bullock’s orioles hybridize extensively along riparian corridors through the Great Plains of North America where they are now in secondary contact^24–26^. Based on an argument for extensive hybridization and the presence of viable hybrid offspring^24^, the two taxa were temporarily lumped into a single species, the ‘Northern Oriole’^27^. Conversely, later studies have suggested the build-up of both pre- and post-mating barriers of species isolation with evidence for some level of assortative mating in the hybrid zone^26,28^ and possible selection driven by divergent climate niches of the parental species, respectively^28,29^. The argument that hybrid orioles suffered from some sort of pre- or post-mating fitness detriment later resulted in an official systematics reversal, splitting these orioles back into their two original taxonomic species^30^.

Despite a long history of study, the few genetic studies of these taxa only characterized general patterns of divergence and introgression between the two oriole species^29,31–34^. Divergence time estimates between the Baltimore Oriole and the Bullock’s/Black-backed Oriole complex of approximately 350,000 years^33^ coupled with an almost 5% mitochondrial divergence^31^ suggests that the two species had previously evolved independently for some time^33^. Earlier studies that utilized fewer markers suggested that the exchange of nuclear regions appeared to be far less restricted than that of the mitochondrial genome^2,33^, indicating ongoing gene flow and possible support for Haldane’s rule. More recently, however, a reduced representation genomic study documented an F_*ST*_ of 0.16 based on 3067 SNPs^34^, indicative of a deeper overall genomic divergence. While there is good evidence for recent and ongoing genetic and phenotypic introgression^34^, we have little understanding of the underlying genomic architecture of potential isolating mechanisms and how these barriers may have accumulated over deeper divergence times. Given the demographic history of these oriole groups, we posit that the coupling of several pre- and post-mating barriers might have worked in conjunction to maintain reproductive isolation. Specifically, based on the research done to date, we predict that selection for divergent plumage traits (pre-mating barriers), as well as for genes conferring putative adaptive functions linked to physiology and behavior (post-mating barriers) may play an important role in shaping the genomic landscape between these species. The Bullock’s-Baltimore oriole hybrid zone thereby presents a tractable system for studying the architecture of barriers to reproduction and how speciation progresses on a genomic scale. Moreover, extensive sampling of genetically and phenotypically diverse individuals across a well-studied hybrid transect provides a valuable data set for evaluating the extent and pattern of differential introgression of neutral and barrier loci across space. Taken together, we present a comprehensive examination of the genomics of accumulating reproductive barriers at mid to later stages of divergence through a combination of whole genome and targeted amplicon sequencing methodologies.

## Results

### The genomic architecture of divergence & introgression in orioles

**Fig. 1:**
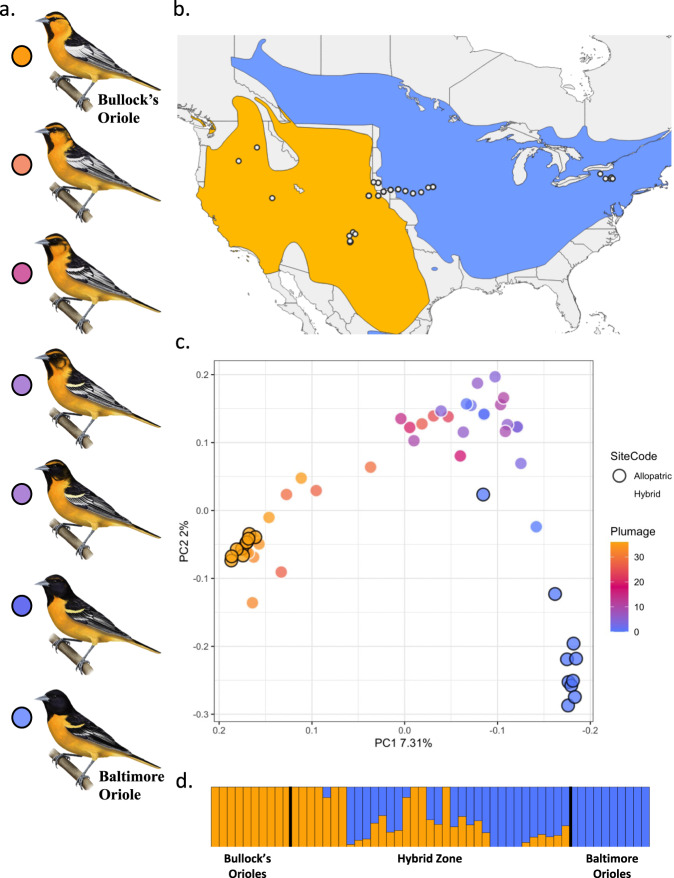
Geographic, phenotypic, and genomic variation in Baltimore and Bullock’s orioles. **a** An example of the phenotypic gradient observed in the two species and their hybrids. Phenotypically pure Baltimore Orioles are in blue and phenotypically pure Bullock’s Orioles are in orange. Illustrations by Megan Bishop, 2019. This image is the property of Cornell University. **b** Sampling locations for the individuals sequenced for this study. The range of both the Bullock’s (orange) and the Baltimore (blue) oriole is shaded on the map. **c** PCA of 55 resequenced individuals based on 11.6 million SNPs. Individuals are colored by plumage score. Black circles indicate individuals sampled outside of the hybrid zone. **d** ADMIXTURE plot (*K* = 2) for re-sequenced males. ADMIXTURE analyses were run using a filtered dataset (110,621 autosomal) that contained no missing data and was pruned to avoid linkage.

Whole-genome resequencing of 55 Baltimore, Bullock’s, and admixed orioles revealed a clinal pattern of divergence corresponding to the phenotypic variation (Fig. 1a, b). Allopatric Baltimore and Bullock’s orioles split along the first PC axis (7.31% of the variation explained) and phenotypically intermediate individuals fell out in the center of the PCA space (Fig. 1c). This gradient of genotypes is consistent with the F1/F2 and backcrossed individuals chosen for sequencing (see methods). Differentiation between allopatric Baltimore and Bullock’s orioles was further supported by a genome wide F_*ST*_ estimate of 0.19. Genome-wide divergence between backcrossed Bullock’s and Baltimore individuals (see methods) was moderately reduced (F_*ST*_ = 0.12) compared to that observed between populations outside of the hybrid zone.

**Fig. 2:**
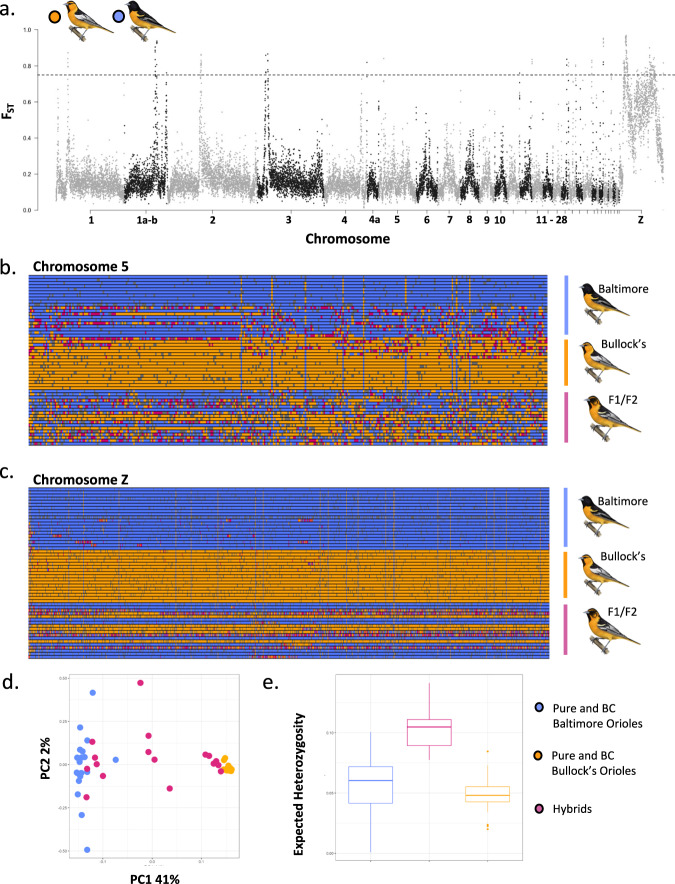
The genomic architecture of divergence between Baltimore and Bullock’s orioles. **a** genome-wide *F*_ST_ estimates between allopatric Baltimore and Bullock’s orioles in 50 kb windows. Dashed line represents the 99^th^ percentile of the mean (*F*_ST_ = 0.75). **b** Genotype plot for chromosome 5 shows an example of admixture patterns for fixed SNPs across the chromosome. Blue represents homozygotes for the reference allele, orange is homozygotes for the alternate allele, and heterozygotes are in pink. Colors are polarized using a Baltimore Oriole. **c** Genotype plot for the Z chromosome shows an example of admixture patterns for fixed SNPs. Blue represents homozygotes for the reference allele, orange is homozygotes for the alternate allele, and heterozygotes are in pink. Colors are polarized using a Baltimore Oriole. **d** PCA of SNPs associated with the larger elevated MDS region (as shown in the dashed black lined box). The 55 individuals sort into three clusters with the ends representing putative homozygotes for the inversion and the middle representing individuals that are putative heterozygotes for the inversion orientation. Individuals are color coded as allopatric/backcrossed for either parental species or hybrid (for this analysis, the hybrid category corresponds only to F1/F2 individuals). Genotypic classes are based on reduced representation sequencing data (see methods). **e** As predicted, heterozygosity for putative heterozygotes for the inversion is higher than that observed in homozygous individuals. Heterozygosity estimates were calculated for individuals assigned to each of the three clusters based on PCA results from panel **c**. Color coding for panel e is the same as that in panel **d**.

Based on our criteria, we observed 203 elevated windows between allopatric populations of Baltimore and Bullock’s orioles (Fig. 2a). If we combine autosomal chromosomes with the Z and retain any window exhibiting a mean F_ST_ estimate greater than the 99^th^ percentile of the mean, in aggregate these divergent windows are a very small fraction of the entire genome (~1%). However, genome-wide patterns of divergence differed substantially between the autosomes (0.37% of the autosomal genome is elevated) compared to the Z chromosome (9.2% of the Z is elevated). Based on variant discovery in allopatric Baltimore and Bullock’s orioles, we additionally selected 150 ancestry-informative markers (melanin-linked, background, fixed, and inversion-linked; Supplementary Fig. 1) for genotyping in 312 additional pure and admixed individuals sampled across the Platte River transect and discuss those patterns in greater detail below.

Consistent with the PCA, ADMIXTURE supported strong assignment of allopatric and backcrossed individuals to either Baltimore or Bullock’s oriole clusters (Fig. 1d). The average ancestry value for putative F1/F2 hybrids was 0.46, supporting previous findings that introgression is recent and ongoing in this system. ADMIXTURE analyses done in groups of 100,000 SNPs across the genomes of the putative F1/F2 hybrids revealed the complexities of introgression on a genomic scale, with individuals exhibiting variable patterns of admixture (Supplementary Fig. 2). A comparison of ADMIXTURE proportions across autosomes versus the Z chromosome also showed an apparent reduction in hybridization across the Z windows, with only a few putative F1/F2 individuals exhibiting admixture on the Z and some individuals exhibiting admixture across most of the chromosome except for the Z (Supplementary Fig. 2). On a finer scale, we plotted genotypes for the 55 individuals using a filtered data set containing only fixed SNPs (defined as having an *F*_ST_ = 1) across each of the chromosomes (Supplementary Fig. 3). Across the autosomes, putative F1/F2 individuals appear to be uniformly admixed, with a relatively high proportion of heterozygous genotypes across most of the genome (Fig. 2b, Supplementary Fig. 3). We observed less mixing on the Z chromosome, with putative F1/F2 hybrids exhibiting clear patterns of uniform homozygote reference, homozygote alternate, or heterozygote genotypes across the entire Z, as opposed to the mosaic of genotypes observed across the autosomes (Fig. 2c, Supplementary Fig. 3).

Due to the pronounced pattern of divergence across the Z chromosome, we investigated the possible presence of a substantial chromosomal inversion. Using a window-based analysis of local population structure in lostruct, we identified two probable clusters of multidimensional scaling (MDS) outliers across the Z (Supplementary Fig. 4) containing a total of 6800 variants. In a PCA of the larger outlier region, we find distinct clustering of the 55 orioles into three discrete groups, which we hypothesize corresponds to homozygotes for the two inversion orientations and heterozygotes (Fig. 2d). PC axis 1 explains 41% of the variation and as anticipated, heterozygosity was higher in the middle cluster, which putatively represents heterozygotes for the inversion (Fig. 2e). While the clustering pattern was less defined with the putative inversion-linked SNPs in the AmpSeq panel, none of the females from the larger data set fell out in the center of a PCA of inversion-linked amplicons (Supplementary Fig. 5). As predicted for a Z-linked inversion, only males can be heterozygotes for the rearrangement. We also note here, that among the amplityped males that were intermediate in the inversion-linked PCA, 82% of the individuals for which we also had hybrid classifications (18 of 22) were assigned as F1 hybrids. The remaining four males that fell out in the approximate center of the inversion-linked PCA were classified as Bullock’s or backcrossed Bullock’s orioles. Taken together, these results are compelling and describe patterns of localized genomic structure that are consistent with inversion expectations. Results from LUMPY offer additional support for a large inversion on the Z chromosome, spanning 43,669,219 BP (from positions 1,747,309 to 45,416,528 on the Z chromosome). After filtering out duplicates and uncharacterized loci, we identified 361 genes located within this putative inversion. We found significant enrichment of genes associated with eye photoreceptor cell differentiation, retina morphogenesis, and metabolic processes, among other biological pathways.

### Pleiotropic interactions among several melanin genes define divergent phenotypes

Phenotypic signals can function as pre-mating barriers to hybridization. We assessed the underlying genomic basis of plumage traits that distinguish Baltimore and Bullock’s orioles. Genome-wide association mapping for eight plumage traits identified several genes associated with each trait, but the distribution and number of outlier SNPs identified by the GWAS differed by plumage trait. We identified significant SNPs associated with all eight traits, including 310 associated with the ear, 2050 associated with the forehead, 279 associated with the greater coverts, 443 associated with the lesser coverts, 79 associated with the neck, 10823 associated with the tail base, 30 associated with the tail tip, and 6515 associated with the throat. In all cases, a large proportion of the outliers identified by the GWAS were on the Z chromosome (on average 90% of the outliers for all traits were on the Z). Of the 20529 significant SNPs identified above, 3438 (17%) were shared by two or more plumage traits. From the GWAS outliers, we identified 56 candidate genes putatively linked to melanogenesis (Supplementary Table 3). We identified genes linked to both multiple and single plumage traits within the orioles, including some strong candidates with several previous links to melanic plumage (i.e., *TYRPI*).

**Fig. 3:**
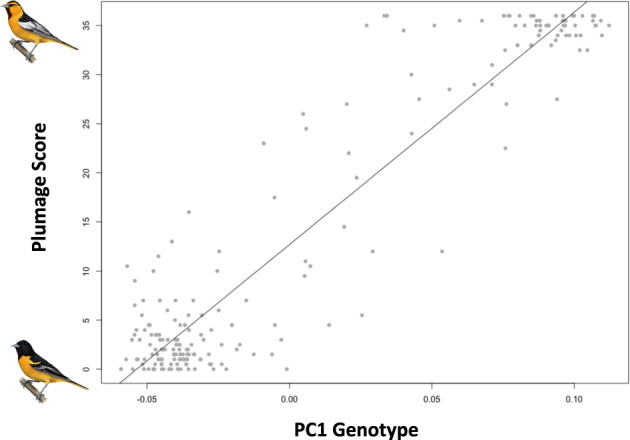
Correlation between genotype and phenotype in Baltimore and Bullock’s orioles. PC1 for genotype (x axis) represents the first axis of a PCA based on amplitypes from candidate melanogenesis genes for all transect individuals. Corresponding plumage scores are on the y axis.

In the larger data set of amplityped individuals, we observed a strong overall correlation between genotypes for the melanogenesis candidates (summarized as PC scores along axis 1) and overall plumage score (*R*^*2*^ = 0.89, *P* < 0.0001, *n* = 206 individuals; Fig. 3). To assess whether pleiotropic interactions among melanogenesis candidates are responsible for plumage variation, we ran several additive and interactive regression models to test for co-dominance and epistasis among melanin amplicons. Excluding single models, we ran 138 regression models assessing the significance of interaction/additive effects. Across all nine plumage traits, 81 of the models (59%) supported a significant additive effect between amplicons on different chromosomes and the score for the trait being tested. 56 (41%) of the regression models supported a significant interactive (epistatic) effect between amplicons and plumage trait; of these, 46 of the 56 models included an interactive effect between melanin candidates on the Z and another region. Six of the twelve most significant models (<0.0005) included interactive effects between a region on chromosome 7 containing the gene *VIM*, which has a putative role in intracellular pigment mobilization. Three of these top models also included interactive effects between *SLC1A2* (linked to skin color variation in other vertebrates^35^) and other regions and three models also included interactive effects between *LVRN* (linked to phaeomelanin pigmentation^36^) and other candidate amplicons.

### Concordant and coincident clines suggest coupled barriers to hybridization

**Fig. 4:**
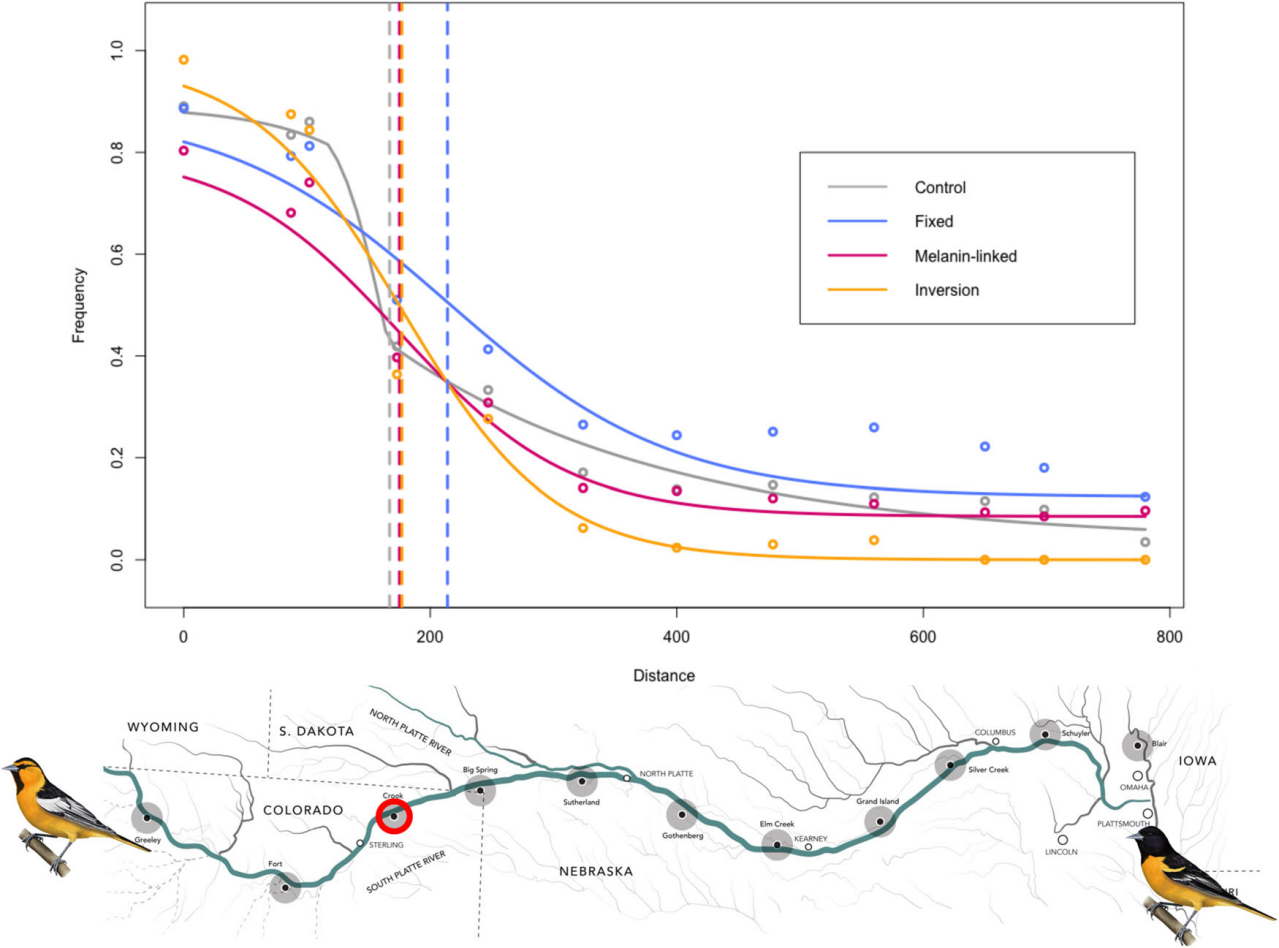
Genome-wide patterns of introgression between Bullock’s and Baltimore orioles. Geographic clines for 4 marker types (top): control (ddRAD sequencing data set from Walsh et al., 2019), fixed amplicons, amplicons putatively linked to melanogenesis, and inversion-linked amplicons. Individuals were sampled along the Platte River transect (bottom) and the clines run from west to east. Crook, Colorado is circled in red and is the approximate center of the control, melanin-linked, and inversion-linked clines. Map inset illustrated by Megan Bishop, 2018.

Geographic cline analyses revealed concordant and coincident cline estimates for three of the four categories of amplicons: putative melanogenesis candidates (175 km for cline center, CI for estimate: 118–219; approximate location Crook), fixed amplicons (214 km center, 172–262; between Crook and Big Springs), and inversion-linked amplicons (177 km center, 152–200; Crook; Fig. 4). Cline estimates for these amplicon-types were also concordant and coincident with a reference “control” cline generated from reduced representation sequencing data (167 km center, 110–198; see methods), supporting comparable selection patterns across the genome. While confidence intervals overlapped among the marker types above, the cline for fixed amplicons was notably shifted to the east (Fig. 4). Allele frequencies were not sufficiently differentiated between Bullock’s and Baltimore orioles (allopatric and admixed individuals) using the background SNPs to generate reliable cline estimates, and this is expected given the low levels of divergence characteristic of background amplicons. Estimates for cline width were: 102 km (8.02–227) for the control cline, 275 km (143–521) for the melanin-linked amplicons, 362 km (210–608) for the fixed amplicons, and 244 km (189–320) for the inversion-linked amplicons. In all cases, there was a longer tail of introgression into Baltimore Orioles to the east, indicative of the westward movement of this zone and overall temporal instability (Fig. 4).

## Discussion

A combination of whole genome and targeted sequencing in Baltimore and Bullock’s orioles revealed a hybrid zone maintained by a complex interaction between multiple putative pre- and post-mating isolating mechanisms. This build up, or coupling, of barriers is perhaps unsurprising given that the species are not sister taxa, however, the buildup has likely been rapid given the divergence time (~350,000 years). Regardless of timing, this system offers empirical data and new perspectives into later stages of divergence along the speciation continuum. Here we discuss the possible mechanisms driving the genomic architecture of divergence between Baltimore and Bullock’s orioles and some implications of these complex interactions for avian hybrid zones more broadly.

A striking pattern in the genome-wide comparison of Baltimore and Bullock’s orioles was the highly differentiated region on the Z chromosome, which is indicative of a large inversion on this sex chromosome. Chromosomal inversions act to suppress recombination when paired with the alternate orientation, and inversions on sex chromosomes, in particular, have frequently been implicated in increased divergence between hybridizing lineages^37–39^. Deleterious mutations and gene combinations typically accumulate more rapidly on sex chromosomes compared to the autosomes, thus structural inversions on sex chromosomes may act as effective barriers to reproduction and are thought to play an important role in the early stages of speciation^40,41^. This pattern holds true in birds, with observed genomic differentiation being much higher on the Z compared to autosomes across several avian hybrid zones^38,42,43^, supporting the potentially important role of structural inversions as a common isolating barrier. Within the oriole data set, we identified a putative inversion on the Z spanning approximately 44 million base pairs and containing 361 genes. We posit that an inversion of this magnitude likely plays an important role in maintaining the oriole hybrid zone. In fact, of the amplityped male individuals that were identified as putative heterozygotes for the inversion, a high proportion (~82%) were classified as F1 hybrids based on reduced representation sequencing data (see methods). This suggests the potential for strong selection against heterozygotes for the inversion, with later stage hybrids more likely to be homozygous for one orientation or the other. We see some support for this from the mapping of genotypes across chromosomes, where we observed a mosaic of heterozygous and homozygous genotypes across the autosomes compared to notably less admixture across the Z. A gene enrichment test based on these 361 genes revealed several significantly enriched regions including locomotory behavior, retina morphogenesis, and eye photoreceptor cell differentiation. Differential expression of photoreceptors (linked to abundance of photoreceptor type and density of visual pigment in each photoreceptor type) is thought to play a role in both color discrimination thresholds in vertebrates as well as sensitivity of response to color^44–46^. Given the clear links between sexual selection and plumage variation^46,47^, one possible hypothesis is that suppressed recombination in the inversion is maintaining gene complexes responsible for visual sensory perception of divergent plumage types in orioles. This link between phenotype and a putative visual preference gene has been previously documented in carrion and hooded crows^21^, suggesting an important role for this linked selection in maintaining reproductive barriers in avian hybrid zones more broadly. A more thorough investigation into the Z chromosome of this species, via long-read sequencing and targeted sequencing of individuals at the putative inversion, will be necessary to fully understand the role of this region in maintaining hybrid zone dynamics in this system.

In addition to the potential post-mating barriers imposed by a putative inversion, we have documented a highly complex architecture of genes underlying plumage characteristics, which appear to be operating pleiotropically to shape phenotypes in Baltimore and Bullock’s orioles. While high throughput sequencing has led to a rapid acceleration in the identification of candidate genes underlying plumage variation in species with phenotypic variation caused by just one or a few loci^12,19,20^, the association of many genes from different pathways with a single plumage color trait is more challenging to study, though this is likely more common than currently recognized^48^. In Baltimore and Bullock’s orioles, we would predict changes in melanin-based pigmentation; however, while melanin is often under strong genetic control^49^, the underlying mechanisms controlling melanin-based traits are not always simple^48,50^. For example, in laboratory mice, melanic pigment production has been linked to upwards of 150 different genes from melanocortin pathways^51^. Moreover, changes in oriole plumage could be a result of complex interactions between carotenoids and melanin, such as color masking (pigments can obscure each other^48,52^) or functional redundancy (similar colors are produced by multiple processes^51^). In fact, chromatographic analyses of pigments in Orchard Orioles (*Icterus spurius*) suggest that carotenoids present in males are indeed masked by high concentrations of melanin^52^. While highly informative, a past research focus on the role of well-characterized genes such as the *melanocortin-1-receptor* (*MC1R*) or *Agouti* in melanic avian plumage may have overshadowed the genomic complexity of many melanin-based phenotypes^50^. The identification of 64 melanin-candidates in our comparison of Baltimore and Bullock’s orioles further highlights this potential complexity underlying seemingly simple and modular phenotypes. Whereas some of our identified candidates have been implicated in plumage coloration in other avian species, including *DCP2* (chickens^53^), *FST* (Gouldian Finch^54,55^), *SEMA6A* (carrion and hooded crows^56^), and *RGP1* (yellow-shafted and red-shafted flickers^57^), there are many that have been implicated in coloration in other vertebrate taxa but are less well characterized in birds. We also note here, that over half of these melanogenesis candidates are located on the Z chromosome, which again raises important questions surrounding linked selection for coloration genes and genes underlying visual perception pathways that may be maintained within the large, putative inversion. Thus, while the orioles present a complex architecture of reproductive barriers, they offer a compelling system for research into the co-evolution of coloration and visual perception in avian systems.

Previous work with the Bullock’s and Baltimore oriole hybrid zone has also found that the hybrid zone has shifted since it was first studied in the 1950s, with movement estimated between 41–73 km westward over a 60-year period^24–26,29,30,34^. Current hypotheses for this westward shift suggest a possible role for climatic changes in the region and the resulting modification of local habitat^28,29^. Empirical examples of climate-mediated hybrid zone movement are becoming more prevalent^43,58–60^, particularly in the Great Plains region of the United States where this pattern has been observed in hybridizing species spanning a suture zone in this region^61,62^. This movement of the hybrid zone, and perhaps its existence, is also likely to be associated with the extensive (and non-climate-driven) expansion of mature riparian woodlands in the Platte River Valley over the past century, following anthropogenic changes in river flow regimes, fire suppression, and altered grazing practices^63^. This increased documentation of hybrid zone movement in nature aids our understanding of past, current, and future distributions of species, from both evolutionary and conservation perspectives, contributing to our understanding of biogeography^64^. Evidence of this movement is apparent from the genomic data as well, with long tails of introgression extending far to the east of the cline centers of the four different genomic regions (fixed, neutral, melanin-linked, and inversion-linked). This asymmetry in cline expansion may occur for several reasons, including differences in dispersal rates or fitness between species^65–67^, with high rates of dispersal and/or lower fitness for one lineage compared to the other both leading to a potential shift in cline centers^4,65^. Moreover, asymmetrical mating barriers can lead to these patterns of cline expansion, with centers shifting toward the species with reduced reproductive isolation^68^. Curiously, the fixed markers show a notable (though not significant) east-shifted cline center compared to the other three marker types, putting it 37–47 km east of the other cline centers. This difference, which is similar to the distance the hybrid zone has moved, further illustrates the dynamic and complex genetic architecture of this system, suggesting different, and potentially opposing selection pressures in maintaining the oriole hybrid zone. While the melanogenesis and inversion-linked markers may be driving the apparent shift in the hybrid zone, perhaps in response to environmental change^28,63^, the fixed markers may be linked to other mechanisms, such as differences in molt and migration^34^.

These pre-mating (plumage variation) and post-mating (structural rearrangements) barriers are likely indicative of an accumulation and coupling of barriers over time, as indicated by largely concordant and coincident clines. Our estimates of cline width and center varied slightly, but confidence intervals overlapped suggesting concordant and coincident clines, overall, as well as locations consistent with cline estimates based on previous reduced representation sequencing^34^. Genome-wide data provide a new perspective, however, by allowing for the analysis of different genomic regions (fixed, neutral, melanin-linked, inversion-linked, for example) and show limited support for patterns of differential introgression among marker types, perhaps because of differing selective pressures. In later stages of divergence, it is likely that reproductive isolation has accumulated exponentially in orioles as barrier loci continue to couple^69^, in turn, we expect that these aggregated barriers cause a “snowball effect” on hybrid fitness^70–72^. While the divergence time between Bullock’s and Baltimore orioles can be classified as relatively recent (<1–2 million years)^73^, the level of genome-wide divergence suggests a steady accumulation of barriers over this time. Data on avian hybrid zones suggest a wide range of levels of reproductive isolation between taxa diverging less than 2 million years ago^73^, with the accumulation of a few strong barriers to reproduction in crows separated by 9,000 years^73^ to detectable levels of introgression between woodcreepers separated by 2.5 million years^23^. Our findings of several barriers, genome wide, despite ongoing introgression between orioles further supports previous findings that the buildup of reproductive isolation, and the resulting genomic signatures, is complex. The level of genome-wide divergence observed between Bullock’s and Baltimore orioles over the estimated time since divergence warrants the revisiting of hybrid fitness in the zone of sympatry and a reassessment of the ultimate impacts and consequences of admixture in these populations.

## Methods

### Sample collection

Baltimore and Bullock’s orioles were sampled during the spring/summer (May 19 through June 27) of 2016–2018 from 12 localities along the Platte River in Colorado and Nebraska, USA^34^. Field work at all sites was approved by local landowners, by Cornell University’s IACUC, and by the US Fish and Wildlife Service at both state (Nebraska permit numbers: 579, 1025, 1091; Colorado permit numbers: 17TRb2328, 18TRb2328), and Federal levels (Federal permit number: MB020189). All specimens collected are currently housed at the Cornell University Museum of Vertebrates. This intensive sampling effort resulted in the collection of 313 vouchered specimens of phenotypic Bullock’s Orioles, phenotypic Baltimore Orioles, and phenotypic hybrids from within the contact zone. From this collection, we sequenced the genomes of 60 male orioles from five genotypic classes, including allopatric Baltimore Orioles (*n* = 10), allopatric Bullock’s Orioles (*n* = 10), backcrossed Baltimore Orioles (*n* = 10), backcrossed Bullock’s Orioles (*n* = 10), and recent generation hybrids (*n* = 20; Fig. 1a; Supplementary Table 1). The hybrid classification of these individuals was based on previous analyses using a subset of fixed SNPs in allopatric/parental individuals obtained from a reduced-representation sequencing study^34^. In short, classification of individuals to genotypic class was based on a combination of hybrid index and interspecific heterozygosity from the ddRAD data. We chose to sequence individuals covering a range of hybrid classes to capture as much genetic and phenotypic variation among admixed individuals as possible. For allopatric populations, we acquired tissues from other museums as well as through our own collecting efforts. We additionally chose 312 individuals, both males and females, for the targeted sequencing of ancestry informative markers (see below for details; Supplementary Table 2).

### Whole-genome sequencing

Genomic DNA was extracted from all samples using the DNeasy blood and tissue kit (Qiagen, CA, USA). DNA concentrations were quantified using the Qubit dsDNA High Sensitivity Assay Kit (Life Technologies). For whole genome re-sequencing, individual libraries were prepared using the NEBNext Ultra II FS kit (New England BioLabs, MA, USA). We followed manufacturer protocol for concentrations ≥ 100 ng with the following conditions: fragmentation for 12.5 min and bead-based size selection of 400–600 bp (insert size of 275–475 bp). Libraries were sequenced on three Illumina NextSeq lanes at the Cornell Institute for Biotechnology core facility. Library quality was assessed using FastQC version 0.11.8 (http://www.bioinformatics.babraham. ac.uk/projects/fastqc). An average number of 24.5 million reads were obtained per individual. Library preparation for four samples (two backcrossed individuals and two recent generation hybrids) failed (i.e. a low number of reads). These four individuals were removed from the data set for all subsequent analyses.

### Amplicon sequencing

Based on variant discovery in pure Baltimore and Bullock’s orioles (see below), we selected 150 ancestry-informative markers (Supplementary Table 3) for genotyping in 312 additional individuals. We chose the following types of markers: fixed variants (*n* = 30) between Baltimore and Bullock’s orioles, melanogenesis candidates (*n* = 56) identified through a GWAS (see below), outliers strongly associated with the inversion on the Z (*n* = 34; see below), and background variants (*n* = 30) which are presumably not under selection. We chose background variants following Knief et al.^74^ with some modification and define these variants as those falling within the 55–65^th^ percentile of the mean *F*_ST,_ 50 kb away from exons, and unlinked (not within 50 kb of each other). Primers were designed using the primer design tool in Geneious Prime 2020.2.3 to amplify a 100–260 bp region including the variant of interest within the first 150 bp. Adapter overhang sequences were added to the forward (5’-TCGTCGGCAGCGTCAGATGTGTATAAGAGACAG‐(locus specific seq)) and reverse (5’-GTCTCGTGGGCTCGGAGATGTGTATAAGAGACAG‐(locus specific seq)) primer sequences. These allowed for addition of the Illumina Nextera barcodes in a second round of PCR.

We amplified all amplicons for each DNA sample in a single multiplex PCR by preparing a single primer mix containing all 300 primers each at a final concentration of 0.7 uM. First round PCR was performed using the Qiagen Multiplex PCR Plus Kit (206152) with 0.3 ul of the above primer mix and 3–40 ng of DNA in a final volume of 11 ul. Initial denaturation at 95 °C for 5 min was followed with 35 cycles of 95 °C for 30 s, 62 °C for 90 s and 72 °C for 30 s and a final extension step of 68 °C for 10 min. A 10-fold dilution of these products was used as the template for a second round of PCR to add the Nextera i5 and i7 index primers. This second round of PCR was performed in a total volume of 11ul with 2ul of the diluted PCR product, 0.5U Platinum™ Taq DNA Polymerase (ThermoFisher, 10966-034), 1 X PCR Buffer, 1.5 mM MgCl2, 0.2 mM dNTP and 0.5 uM of i5 and i7 primer. Reaction conditions are as follows: initial denaturation at 95 °C for 2 min, followed by 7 cycles of 95 °C for 30 s, 55 °C for 30 s and 72 °C for 30 s. This was followed by 95 °C for 10 s, 55 °C for 3 min (with a 0.1 deg/second ramp), 72 °C for 30 s and a final extension step of 72 °C for 5 min. PCR products were then pooled equally and cleaned using 1:1 ratio of SPRI beads (made using Sera-‐mag SpeedBeads; Fisher Scientific, 09-981-123^75^). This library was then sequenced by the Cornell Institute of Biotechnology Resource Center (BRC) on an Illumina MiSeq instrument (single end 150 bp).

### Data filtering & variant discovery

For the whole-genome data set, we used AdapterRemoval version 2.1.1 for sequence trimming, adapter removal, and quality filtering, requiring a minimum Phred quality score of 20 and merged overlapping paired-end reads. We aligned filtered reads to the Myrtle Warbler Reference genome (*Setophaga coronata coronata*)^76^ using the default settings in BWA 0.7.4^77^ and obtained alignment statistics from Qualimap version 2.2.1^78^. The mean percentage of reads mapped to the reference genome was 85% and the mean coverage across all individuals was 4.1x. We used Samtools version 1.9^79^ to convert all resulting BAM files to SAM files and to sort and index files. We used Picard Tools v.2.19.2 (https://broadinstitute.github.io/picard/) to add index groups and mark duplicates. We used the *Haplotype Caller* module in GATK version 3.8.1^80^ for SNP variant discovery and genotyping for the 56 orioles and used the following filtering parameters to remove variants: QD < 2, FS > 60.0, MQ < 30.0, and ReadPosRankSum < −8.0. We additionally filtered out variants that were not biallelic, had minor allele counts less than 4, mean coverage less than 2X or more than 50X, and more than 20% missing data. This resulted in a total of 11,651,297 SNPs across the five ancestry categories (parental Baltimore oriole, parental Bullock’s oriole, F1/F2, backcrossed Baltimore oriole, and backcrossed Bullock’s oriole). We removed one additional individual (backcrossed Bullock’s Oriole) due to high relatedness with another male in the data set (*r* = 0.53) resulting in a final data set containing 55 orioles.

For the amplicon data, we assessed read quality using FastQC version 0.11.8 (http://www.bioinformatics.babraham. ac.uk/projects/fastqc) and removed adapter sequences using Cutadapt version 2.1^81^. We subsequently removed reads containing at least a single base with a Phred quality score of less than 10 (using fastq_quality_filter, FASTX-Toolkit). We additionally removed sequences if more than 5% of the bases had a Phred quality score less than 20. These filtered reads were then aligned to a reference consisting of only the predicted amplified regions (pulled from the Myrtle Warbler reference genome) using Bowtie version 2.3.5.1 with the *very-sensitive-local* option^82^. We used Samtools version 1.9 to convert all resulting BAM files to SAM files and to sort and index files. Variants were called using bcftools -*mpileup* and *-call*. We removed 31 amplicons due to poor or no amplification. For the remaining 119 amplicons, we outputted only the targeted SNP. This resulted in 120 SNPs (one amplicon contained 2 targeted SNPs). We filtered this dataset to exclude all individuals with a mean depth of less than 2, which removed all negative controls and 8 individuals. Finally, sites with more than 50% missing data were removed and 94 sites (78%) were retained.

### Summary statistics and genomic differentiation

Whole-genome sequences from the 55 male individuals were used to describe the overall landscape of divergence between Baltimore and Bullock’s orioles. First, descriptive statistics, including nucleotide diversity (pi) and Tajima’s D, were estimated for each genotypic class in 50 kb windows using VCFtools^83^. To assess patterns of genetic structure, principle component analysis (PCA) was performed on all genome-wide SNPs using the *SNPRelate* package in R^84^.

We calculated *F*_ST_ in both 50 kb windows and for individual SNPs between allopatric individuals. We additionally calculated *F*_ST_ between backcrossed Baltimore and Bullock’s orioles and only considered putative outlier regions if they were elevated in *both* allopatric and backcrossed comparisons. Here, we assume that regions of elevated divergence purely due to geographic isolation in allopatry will be eroded by gene flow in the hybrid zone. Thus, regions exhibiting elevated divergence in allopatry as well as the hybrid zone are likely important for maintaining reproductive isolation between Bullock’s and Baltimore orioles. For both comparisons, windows were dropped if they contained fewer than 10 SNPs. We defined elevated regions of divergence as any window exhibiting a mean *F*_ST_ estimate greater than the 99^th^ percentile of the mean and containing at least one individual SNP with an *F*_ST_ of 1. Due to patterns of elevated divergence across the entirety of the Z chromosome that were discordant with autosomal patterns, we only applied this outlier detection method to the autosomes.

### Putative inversion on the Z chromosome

A defined region exhibiting elevated divergence between Baltimore and Bullock’s orioles on the Z chromosome was indicative of a putative inversion. Thus, to detect possible structural rearrangements, we used the R package *lostruct*^85^, which detects regions of abnormal population structure that may be caused by inversions (Supplementary Code). To detect patterns of population structure across the genome, principal component analysis (PCA) was applied to non-overlapping windows 100 SNPs in size. For each PCA, Euclidean distances between matrices for the first two principal components were calculated and mapped using multidimensional scaling. This approach provides a measure of how similar patterns of population structure are across the windows. MDS values were plotted along the Z chromosome and outlier MDS windows were defined as those with absolute values greater than the 99^th^ percentile of the mean. Following Huang et al.^86^, we expect that for an inversion, a PCA should divide individuals into three groups with homozygotes representing the two orientations and heterozygotes between the arrangements falling into an intermediate cluster. Based on the methods of Huang et al.^86^, we used *SNPRelate* to calculate PCAs for all SNPs from the putative inversions identified above. Furthermore, based on the expectation that groups identified in the PCA represent heterozygotes and homozygotes for the inversion orientation, we expect higher heterozygosity in the center group. For each putative inversion, variable sites were retained from outlier MDS windows and individual heterozygosity was calculated in VCFtools. We investigated these patterns in the 55 sequenced individuals, color-coding individuals by known genotypic class (allopatric, backcrossed, F1/F2) for visualization purposes. Using the inversion-linked SNPs identified through this process, we evaluated the genotype distribution (proportion of homozygous and heterozygous individuals for the two inversion orientations) across the 312 amplityped individuals.

Lastly, we identified approximate breakpoints for the putative Z chromosome inversion using Lumpy^87^ using information from split and discordant mapped reads. We used the default parameters and included the ten allopatric individuals from each species for this analysis. We then used the R package GenomicRanges^88^ to find overlapping annotations within the Myrtle Warbler reference genome and to extract genes within the inversion and near the putative breakpoints (Supplementary Table 4). Gene enrichment analyses for genes associated with the inversion were performed using Fisher’s Exact tests and multiple testing correction with the Benjamini-Hochberg FDR^89^ in PANTHER^90^.

### Quantifying plumage variation

Given the importance of phenotype as a pre-mating barrier to hybridization, we quantified plumage variation in re-sequenced and amplityped individuals to identify underlying genetic mechanisms of plumage color. We quantified phenotypes of adult males by adapting the classic plumage scoring criteria developed by Sibley and Short^24^. We scored 9 plumage traits in 3 regions of the body: 5 traits in the head (supercilium, forehead, ears, neck, and throat), 2 in the tail (tail tip and base), and 2 in the wing (greater and lesser coverts). Scores for all plumage traits ranged from 0 to 4, where 0 indicated “pure” Baltimore plumage traits and 4 indicated “pure” Bullock’s Oriole traits (the sum of all 9 trait scores ranged from 0 to 36).

### Admixture mapping

To identify the genomic underpinnings of plumage variation in Bullock’s and Baltimore orioles, we employed genome-wide association analyses implemented in the package gemma^91^. gemma detects SNPs that are associated with specified traits while controlling for population structure by including a relatedness matrix in the model. Because gemma requires a complete SNP data set, we imputed missing data using beagle v 4.1^92^. We conducted separate univariate linear mixed models for each of the nine plumage traits and used the Wald test (-lmm 1) with a significance threshold of α-value of –log_10_ = 7 to identify associations between genotype and phenotypes^93^. Models for supercilium failed to converge, and thus we present results for 8 out of the 9 plumage traits. We visualized results using the *manhattan* function in the qqman package in R^94^.

### Pleiotropic interactions among plumage candidates

To validate the 56 melanin candidates included in the amplicon panel, we first evaluated the distribution plumage score for each trait by individual genotype. For each plumage trait, we fitted SNPs with a significant effect on phenotype as factors to test for a co-dominant (additive) or epistatic (interactive) effect. For all models, genotypes were encoded as Baltimore-like, heterozygote, or Bullock’s-like using the–012 option in VCFtools. A series of linear regression models were used to evaluate the contribution of each term in predicting plumage traits. Due to the large number of possible combinations, if putative melanin candidates were found on the same chromosome, we chose one SNP per million base pairs and retained the SNP with the highest *F*_ST_, resulting in 185 models. Linear regression models were run using the base package in R.

### Gene identification

All outlier regions were inspected in Geneious version 9.1.5^95^. For each outlier region, we compiled a list of gene models within 40 kb of each region (flanking either the window for *F*_ST_ outliers or single SNPs for GWAS outliers) and used the R package GenomicRanges to find overlapping annotations within the Myrtle Warbler reference genome. We obtained information on these annotations from the UniProt database (http://www.uniprot.org/).

### Patterns of differential introgression

To characterize general patterns of hybridization between Bullock’s and Baltimore orioles, Admixture v 1.2.3^96^ analyses were run using a filtered dataset (110,621 autosomal) that contained no missing data and was pruned to avoid linkage using the script ldPruning.sh (https://github.com/joanam/scripts/blob/master/ldPruning.sh). Because we were only interested in a signal of admixture between the two species, we investigated two possible population clusters, with 200 bootstrap resampling iterations. We also characterized patterns of introgression across the genome, with a focus on patterns of differential introgression and reduced recombination on the Z chromosome versus the autosomes (Supplemental Code). A VCF containing the 11.6 million SNPs from the whole genome data set was split into individual VCFs per chromosome. Each per-chromosome VCF was than split into smaller subsets, containing 100,000 SNPs per file. We then ran Admixture on these smaller SNP data sets across the entire genome using the same parameters described above. Admixture proportions were plotted across the genome for each of the 20 F1 individuals. We additionally investigated patterns of fixation and heterozygosity for parental alleles across the genome. We filtered each per-chromosome VCF to retain only fixed SNPs (sites with an *F*_ST_ = 1) and visualized genotypes across the genome using the R package GenotypePlot^97^.

To evaluate patterns of differential introgression across the hybrid zone for the four categories of ancestry informative markers, we used the Metropolis-Hastings Markov chain Monte Carlo algorithm employed in the R package *HZAR*^98^ to fit a series of geographic cline models to allele frequencies for each SNP targeted in the amplicons. We estimated clinal transitions of allele frequencies for the genotyped individuals for fixed, background, melanin-linked, and inversion linked SNPs. We fixed the variance and the mean to 0 (Baltimore Oriole) or 1 (Bullock’s Oriole) and fit 3 possible tail models (none fitted, mirror tails, or both tails estimated separately). Because our background SNPs showed little differentiation among species, we additionally included a “control” cline that was generated from Structure Q-values from a ddRAD data set of the same individuals^34^ to provide a proxy for a “neutral” cline. For all clines, we compared confidence intervals for cline width and center to assess whether clines were coincident (overlap in cline centers) and concordant (equal widths).

### Reporting summary

Further information on research design is available in the Nature Portfolio Reporting Summary linked to this article.

## Supplementary information

Supplementary Information

Description of Additional Supplementary Files

Supplementary Data 1

Supplementary Data 2

Supplementary Data 3

Supplementary Data 4

Reporting Summary

## Data Availability

A filtered VCF, associated metadata, and GWAS summary statistics are available on Dryad (10.5061/dryad.kkwh70s80). Raw sequencing data is available in the NCBI short reads archive (BioProject accession number PRJNA923032). Figures and associated data are on figshare: https://figshare.com/projects/A_COMPLEX_GENOMIC_ARCHITECTURE_UNDERLIES_REPRODUCTIVE_ISOLATION_IN_AN_ORIOLE_HYBRID_ZONE/156705.

## Code availability

Scripts and bioinformatic pipelines are included in the supporting document as well as on GitHub: https://github.com/jenwalshemond/WGSScripts. Scripts for main figures in the manuscript are deposited on figshare.
