# Supplementary material for: A complex genomic architecture underlies reproductive isolation in a North American oriole hybrid zone
Source: Commun Biol. 2023 Feb 7;6:154. doi: 10.1038/s42003-023-04532-8 (PMC9902562; doi:10.1038/s42003-023-04532-8)
Supplement: Supplementary file 1 — Supplementary Information [file 42003_2023_4532_MOESM1_ESM.pdf]

A COMPLEX GENOMIC ARCHITECTURE UNDERLIES REPRODUCTIVE ISOLATION IN AN AVIAN HYBRID ZONE

Jennifer Walsh, Shawn M. Billerman, Bronwyn G. Butcher, Vanya G. Rohwer, David P. L. Toews, & Irby J. Lovette

Supplementary Information:

Supplementary Figures:

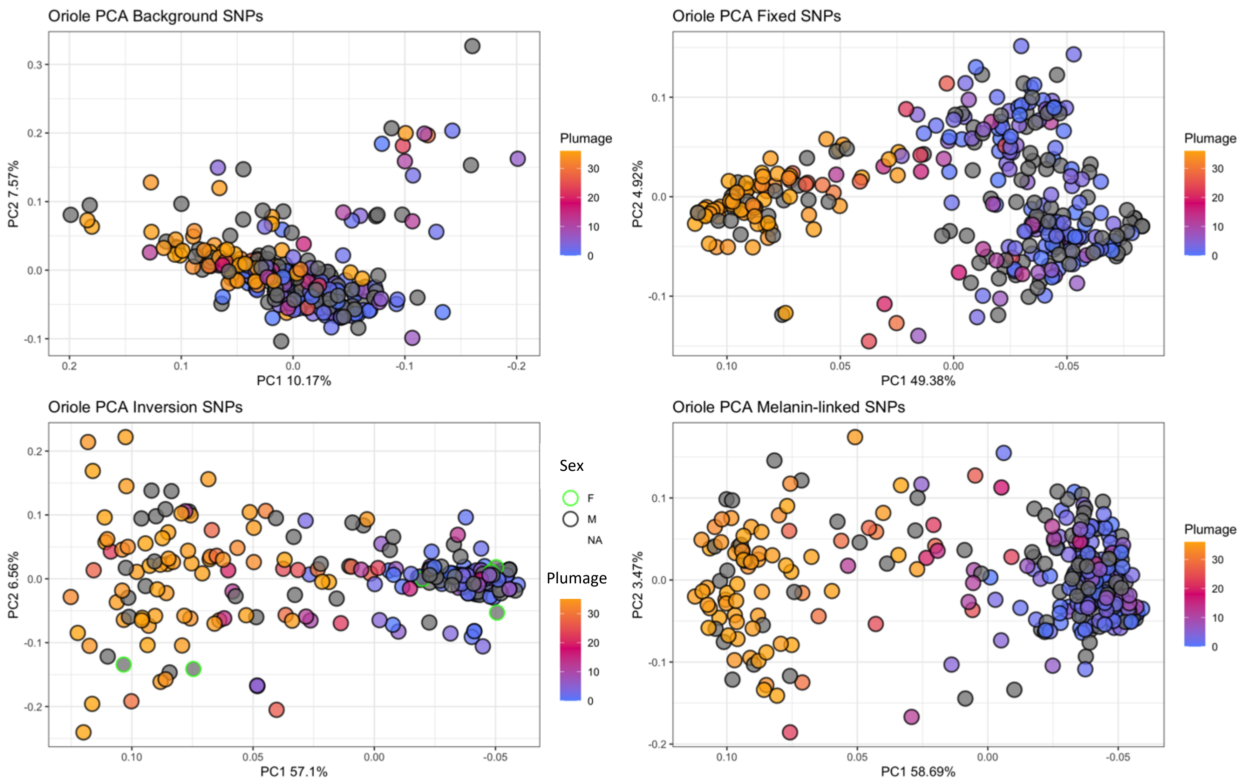

Supplementary Figure 1: PCA of all amplityped individuals based on four categories of SNPs: background, fixed, inversion-linked, and melanin-linked. Individuals are color-coded by plumage color (gray points are individuals with no plumage data available).

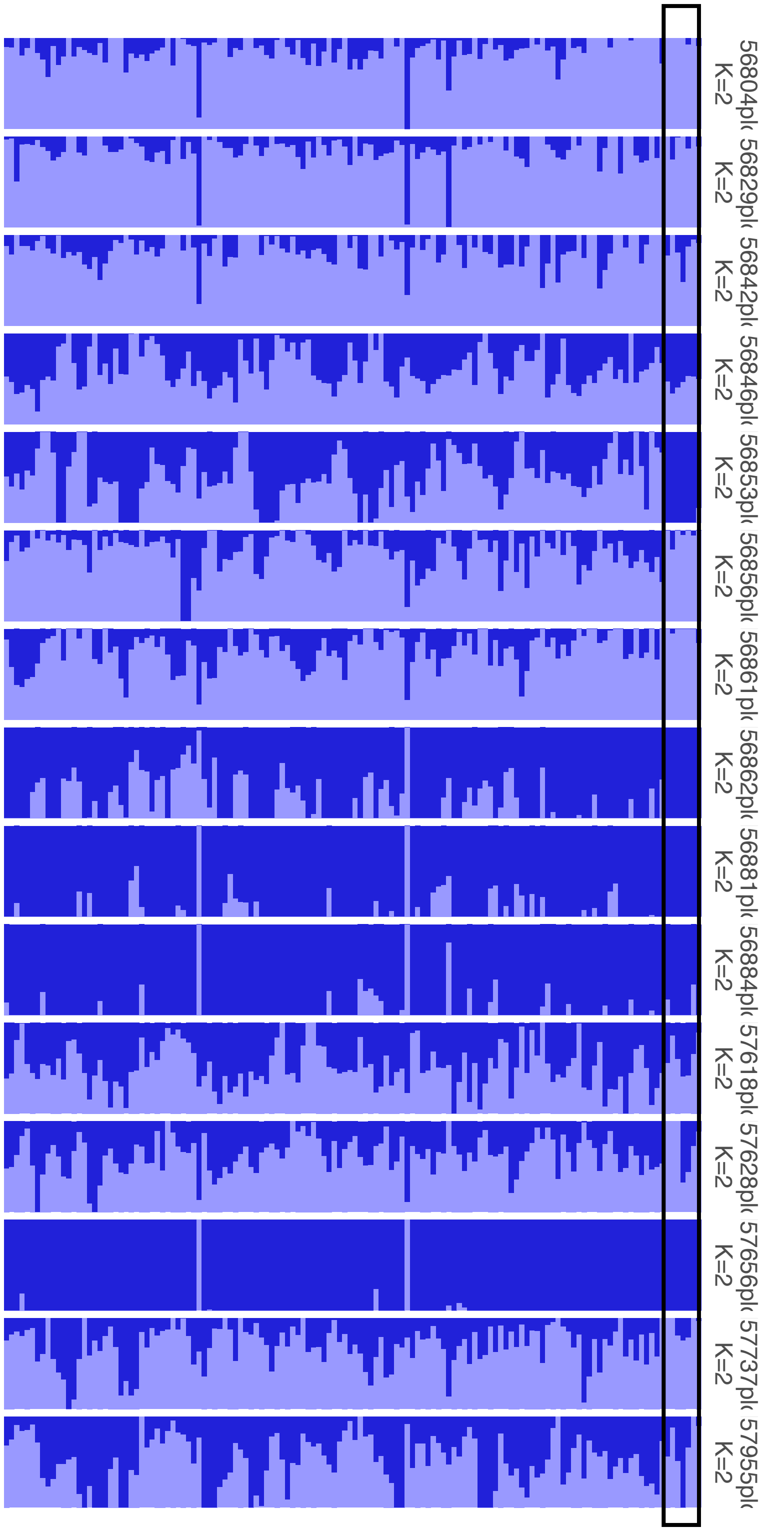

Supplementary Figure 2: Admixture plots for putative F1/F2 individuals. Each plot is for one individual and each column within a plot corresponds to a window containing 100,000 SNPs ordered across the genome. Windows are in order from chromosome 1 (left) to Z (right). The Z chromosome is boxed in to show apparent reduction in introgression across these windows.

Chromosome 1 – Fixed SNPs

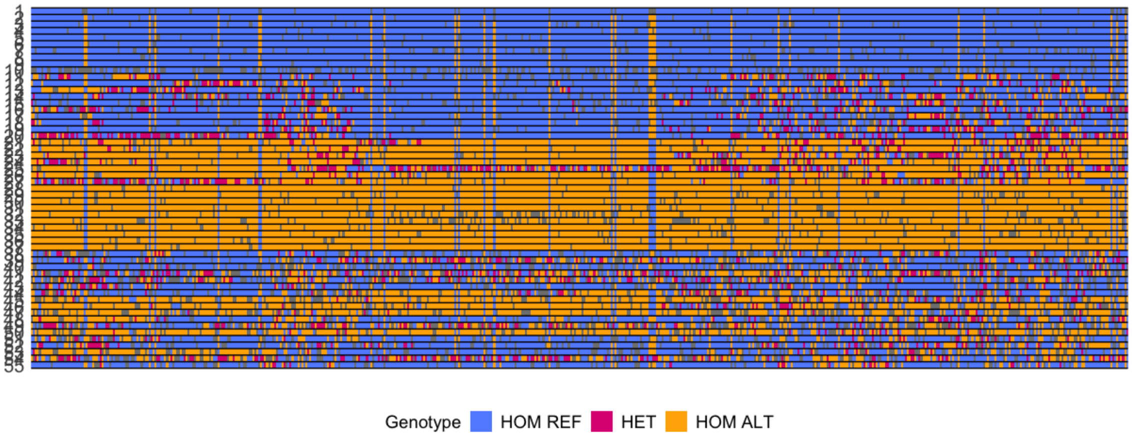

Chromosome 1a – Fixed SNPs

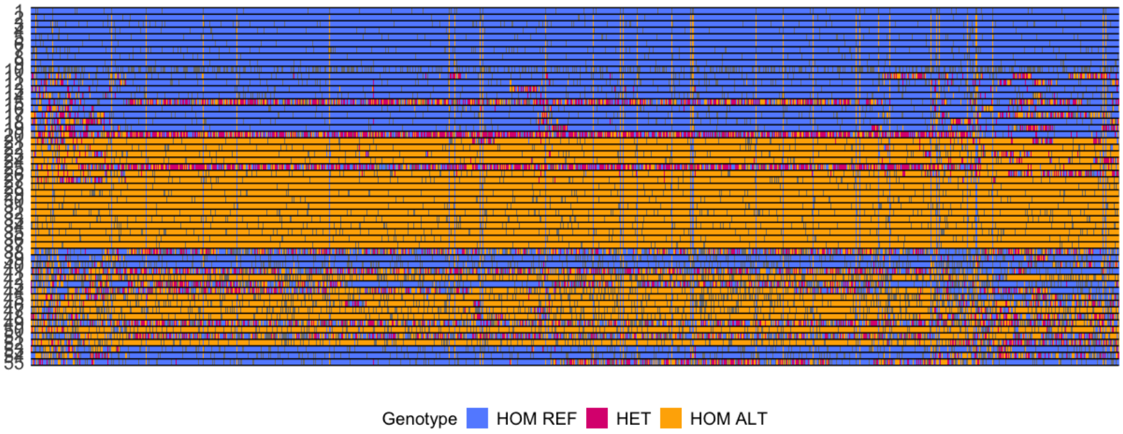

Chromosome 2 Fixed

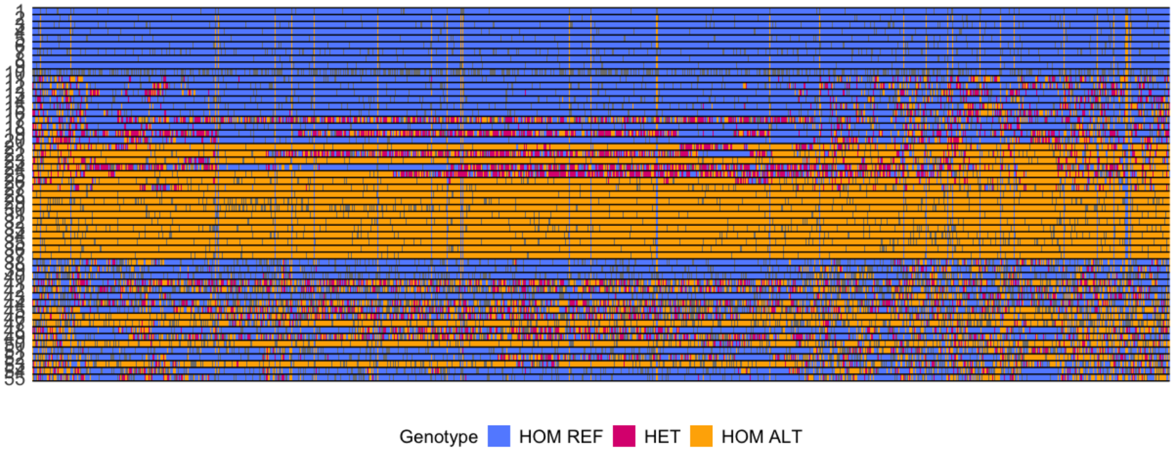

Chromosome 3 Fixed

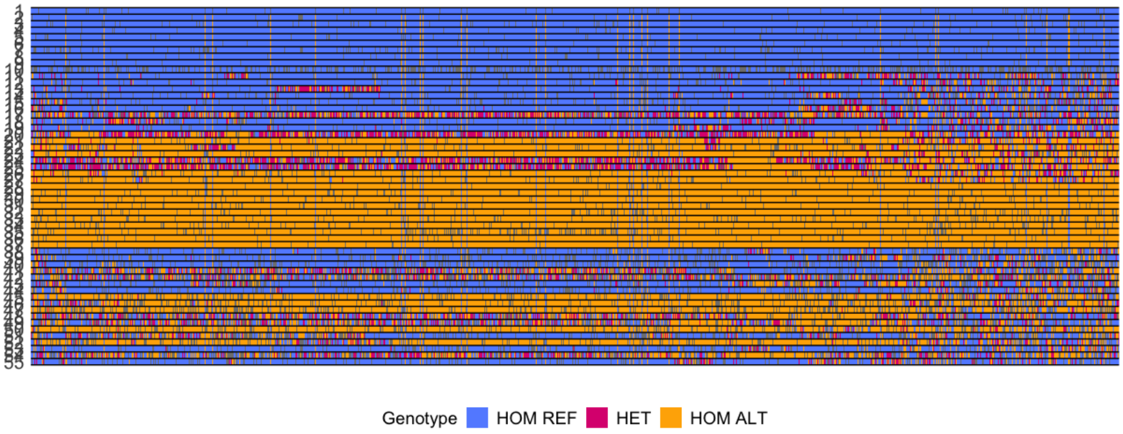

Chromosome 4 Fixed

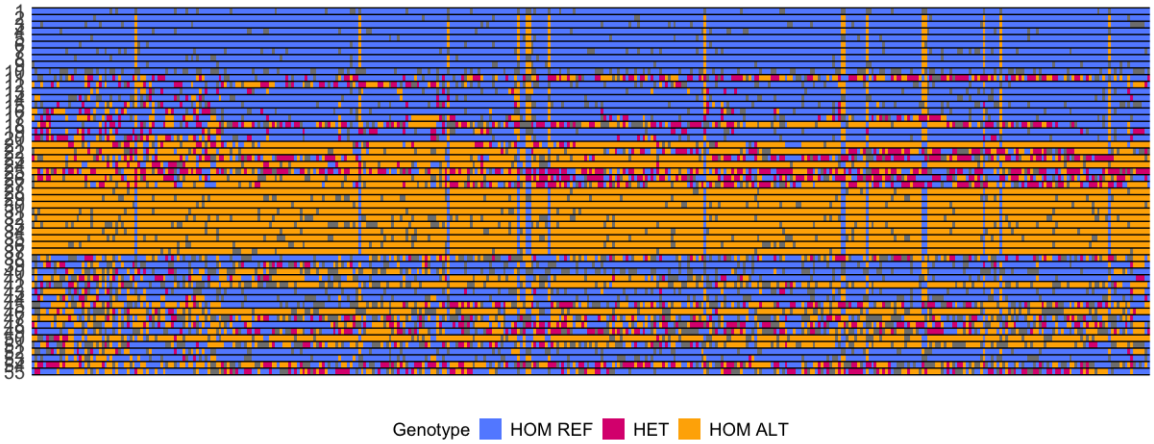

Chromosome 4a Fixed

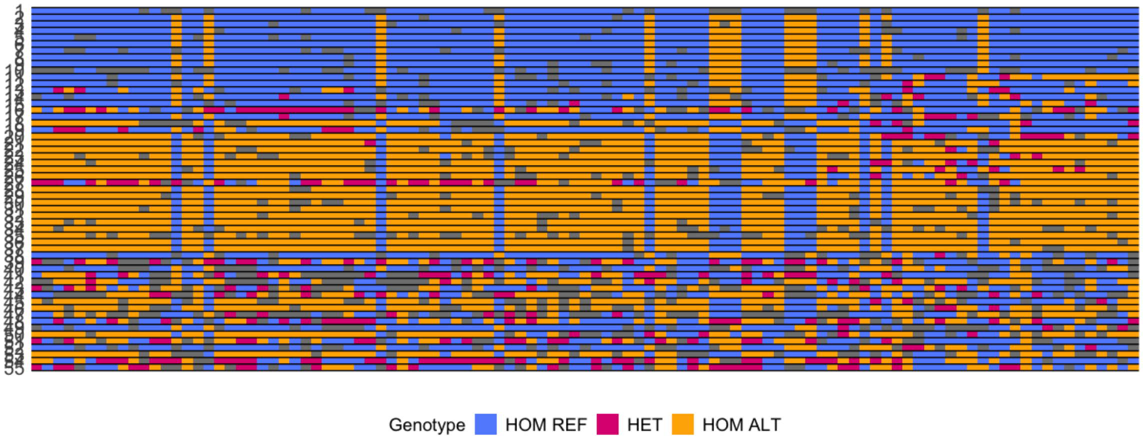

Chromosome 5 Fixed

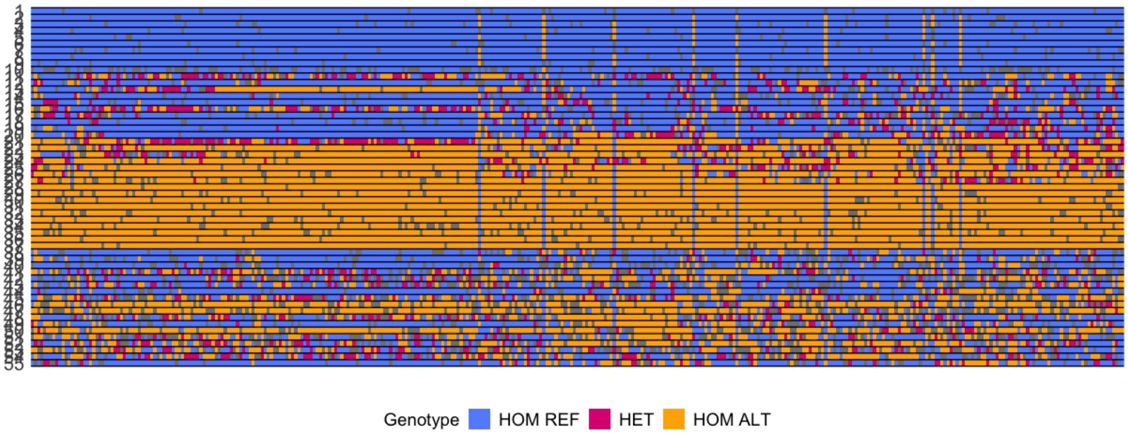

Chromosome 6 Fixed

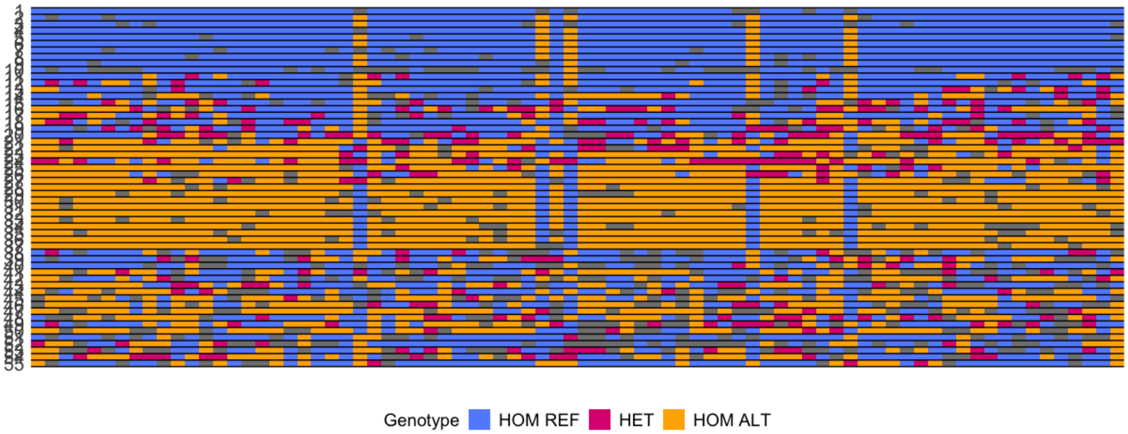

Chromosome 7 Fixed

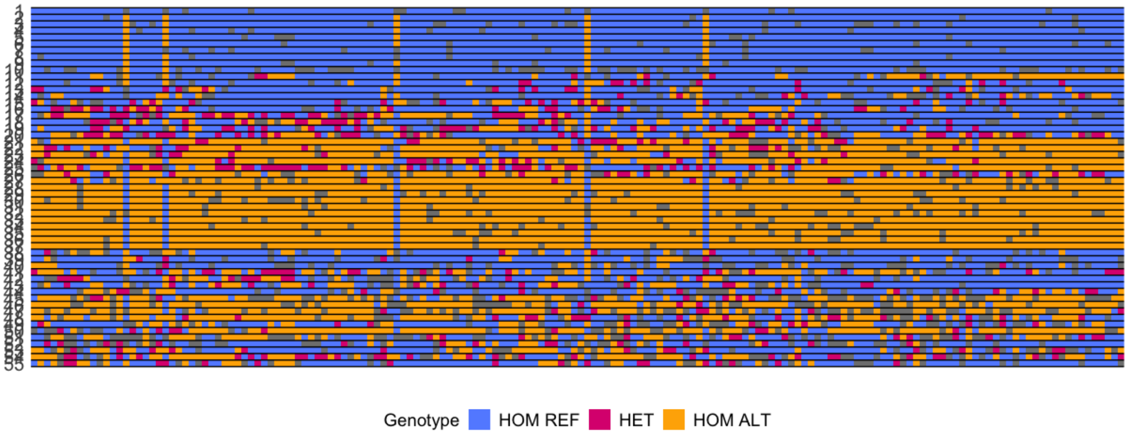

Chromosome 8 Fixed

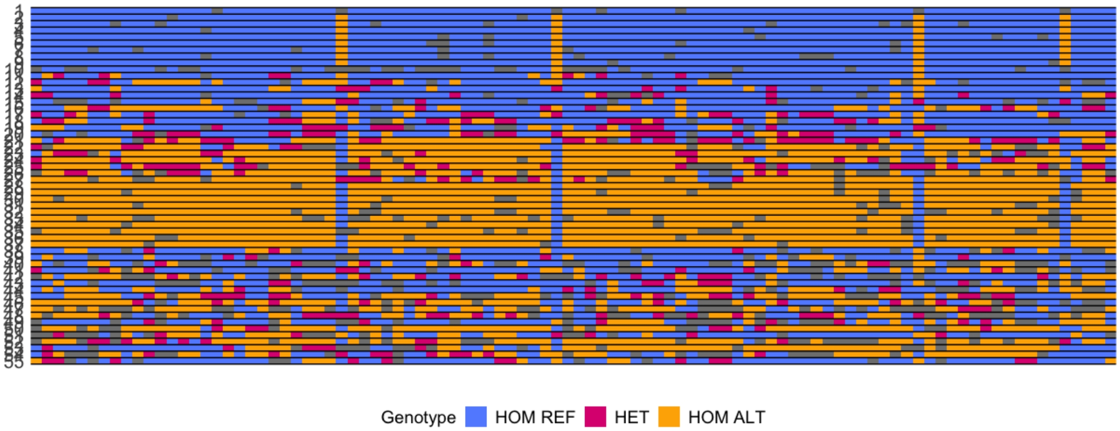

Chromosome 9 Fixed

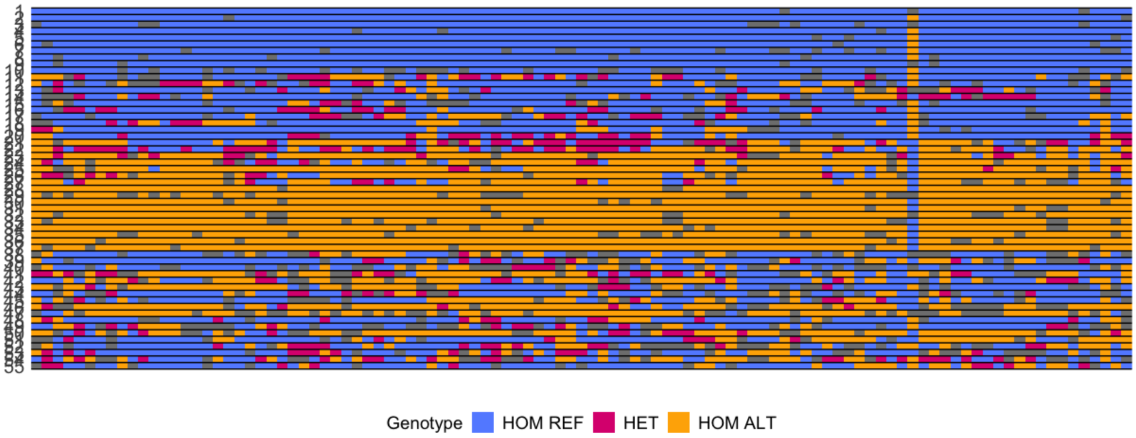

Chromosome 10 Fixed

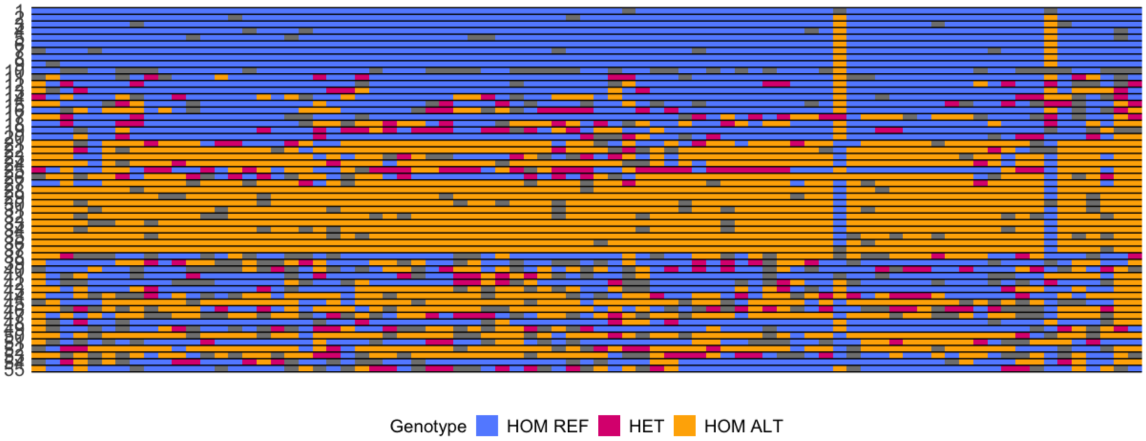

Chromosome 11 Fixed

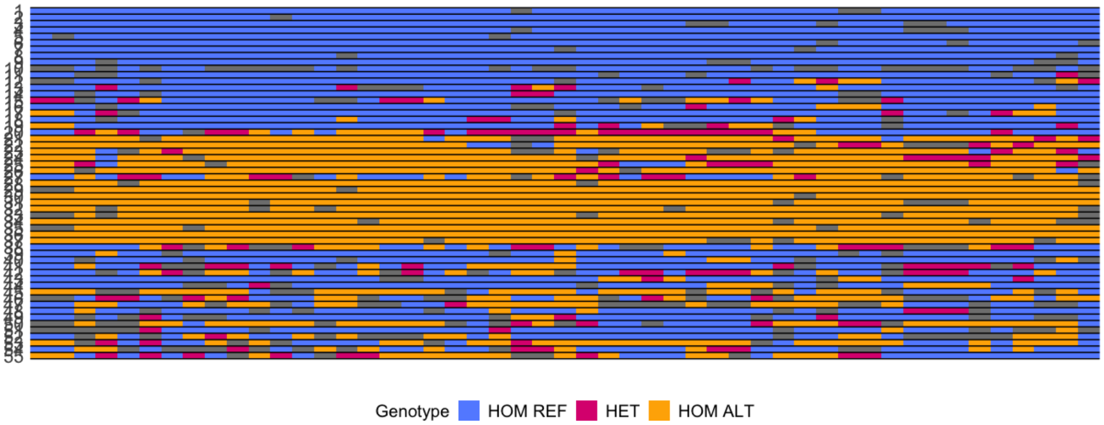

Chromosome 12 Fixed

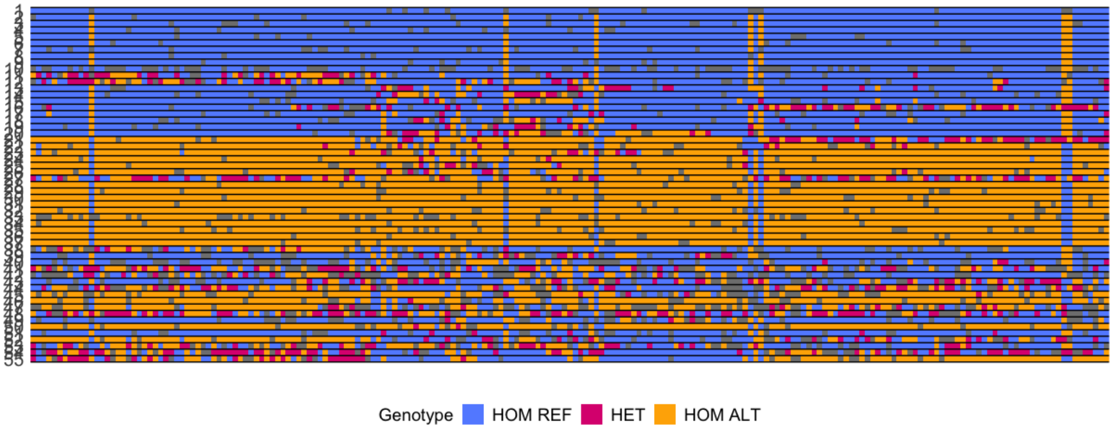

Chromosome 13 Fixed

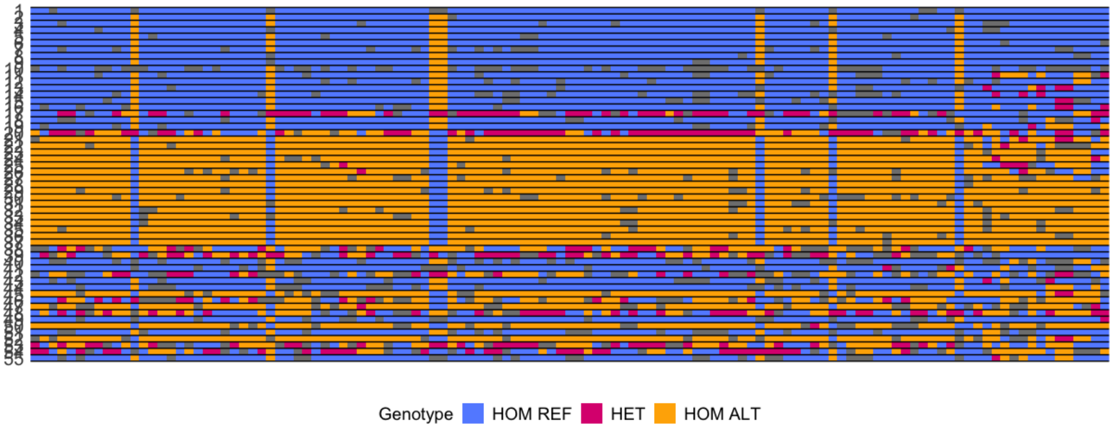

Chromosome 14 Fixed

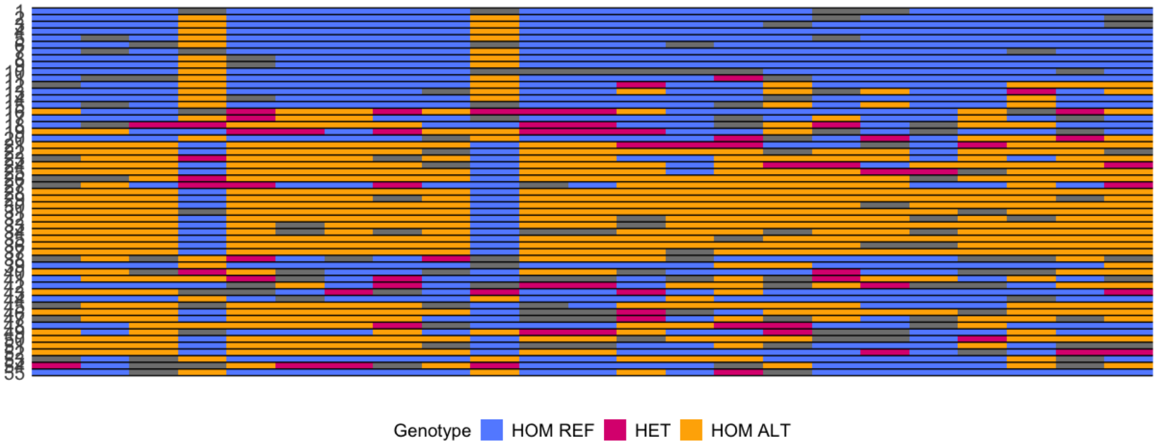

Chromosome 15 Fixed

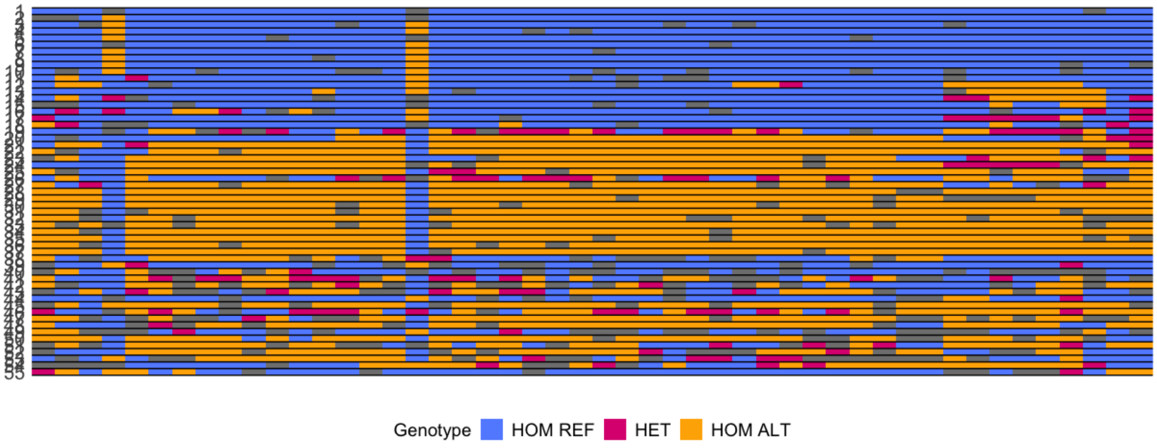

Chromosome 17 Fixed

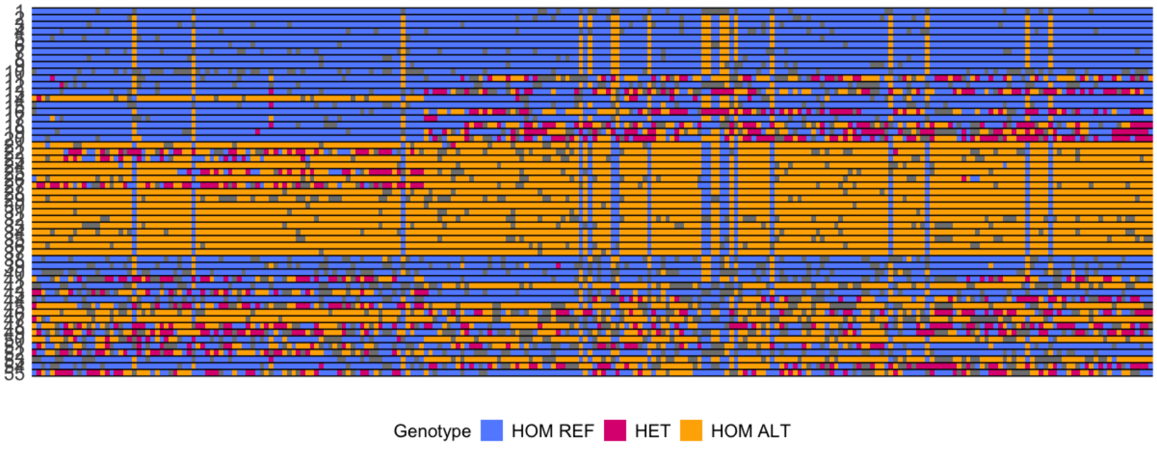

Chromosome 18 Fixed

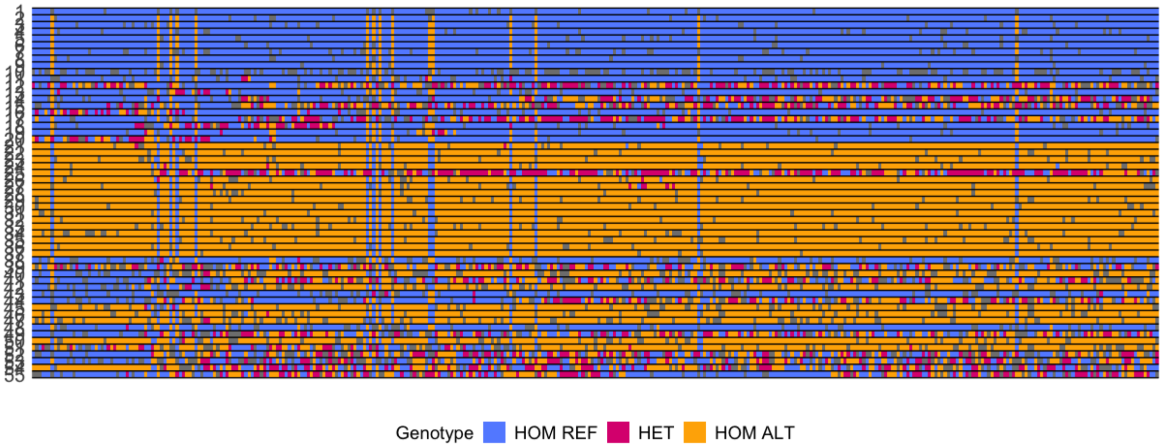

Chromosome 19 Fixed

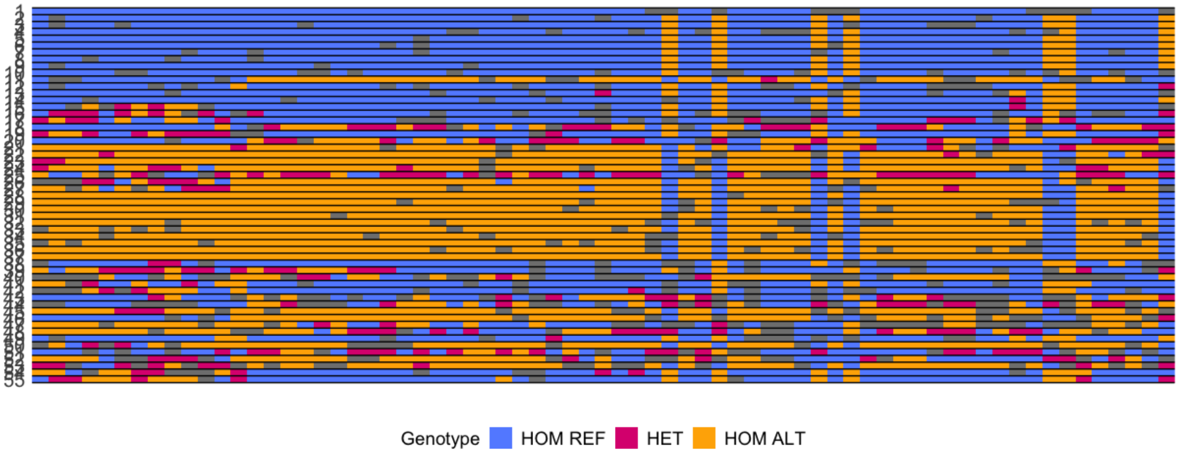

Chromosome 20 Fixed

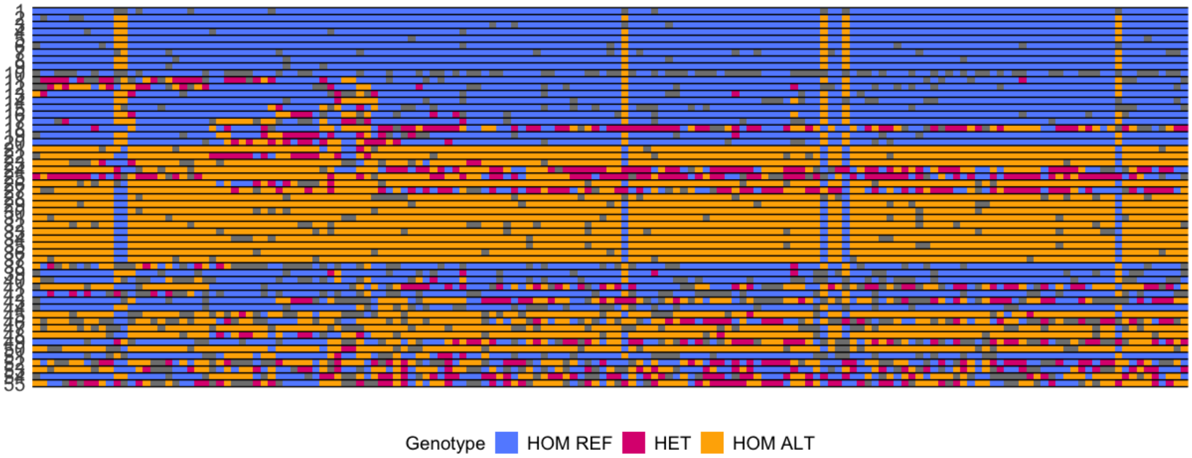

Chromosome 23 Fixed

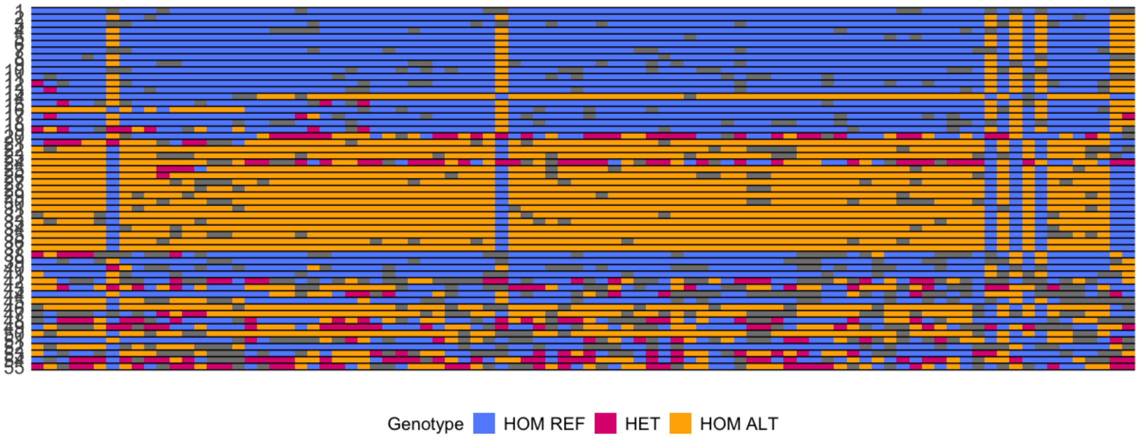

Chromosome 24 Fixed

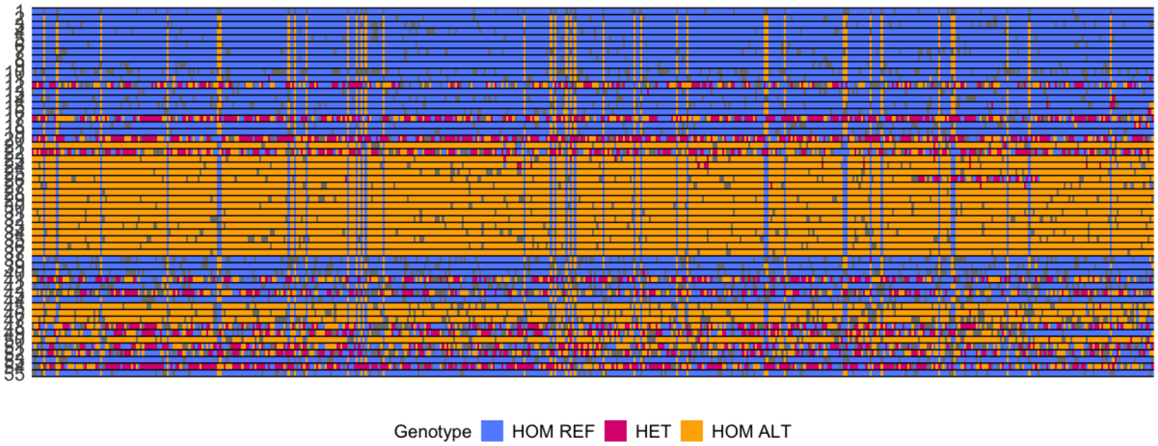

Chromosome 26 Fixed

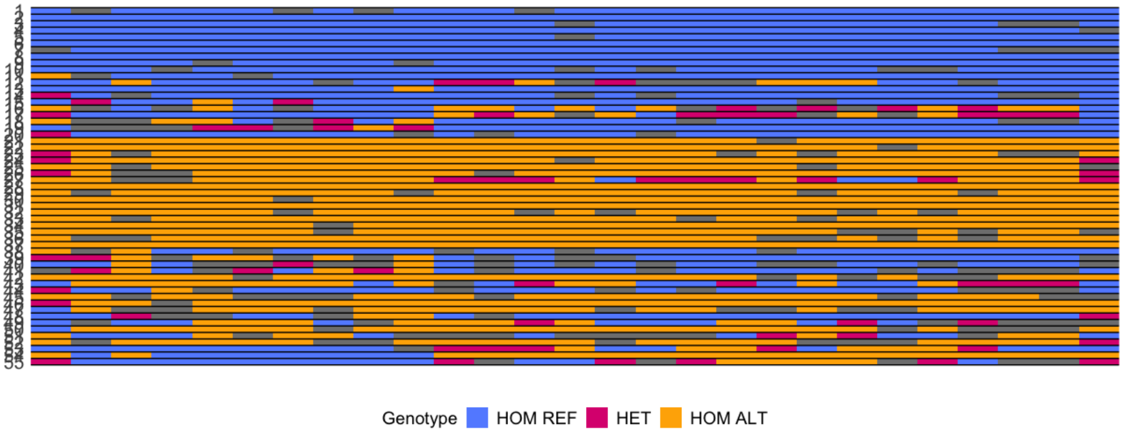

Chromosome Z Section 1 Fixed

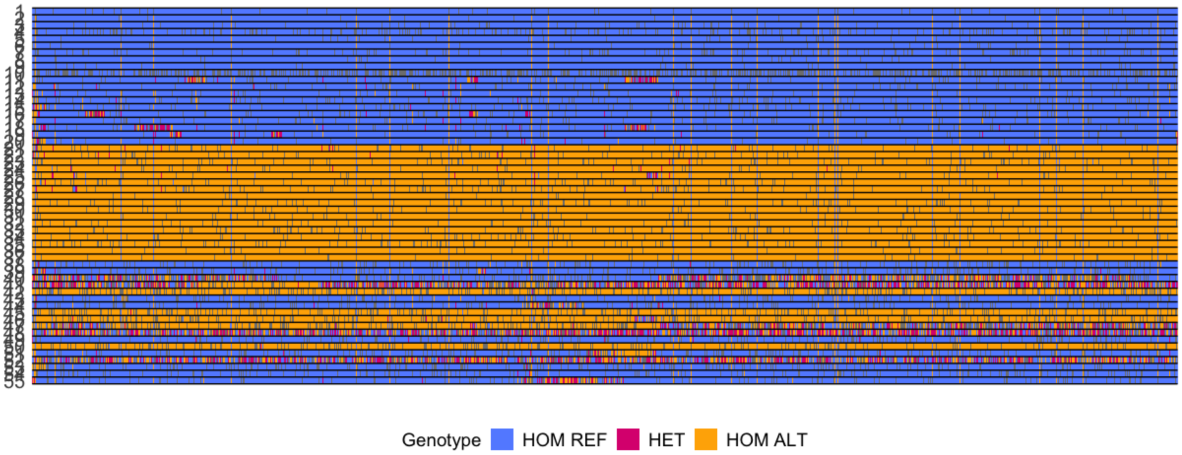

Chromosome Z Section 2 Fixed

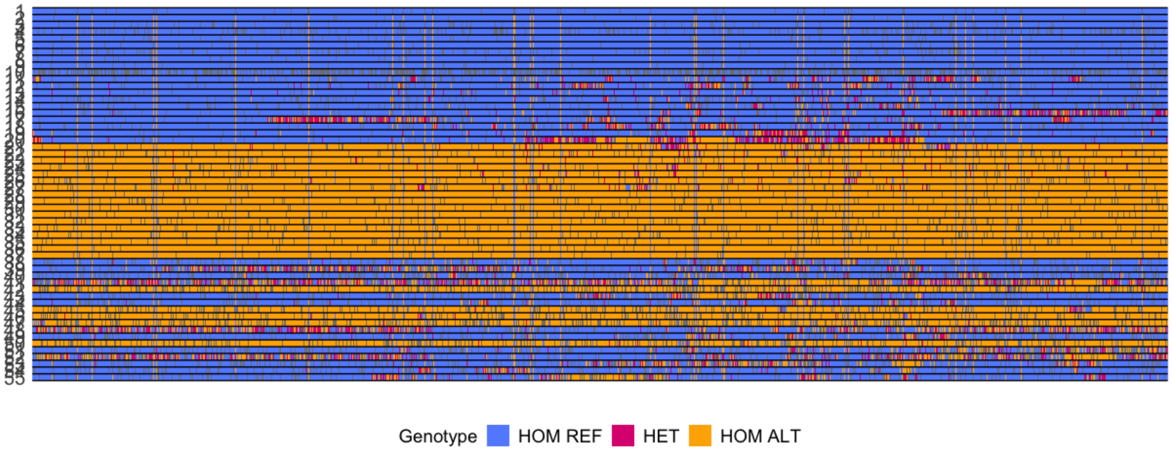

Chromosome Z Section 3 Fixed

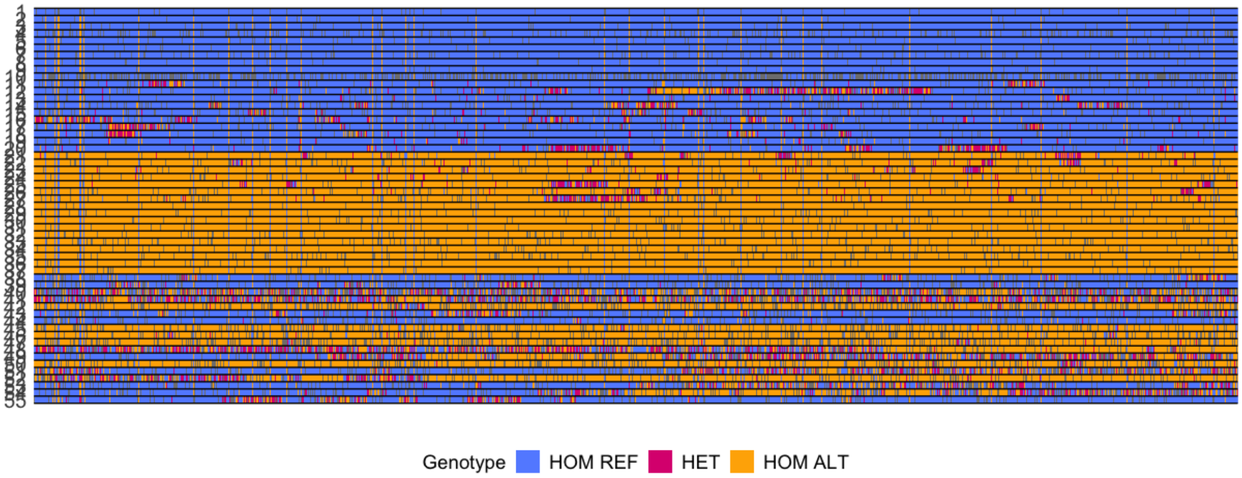

Chromosome Z Section 4 Fixed

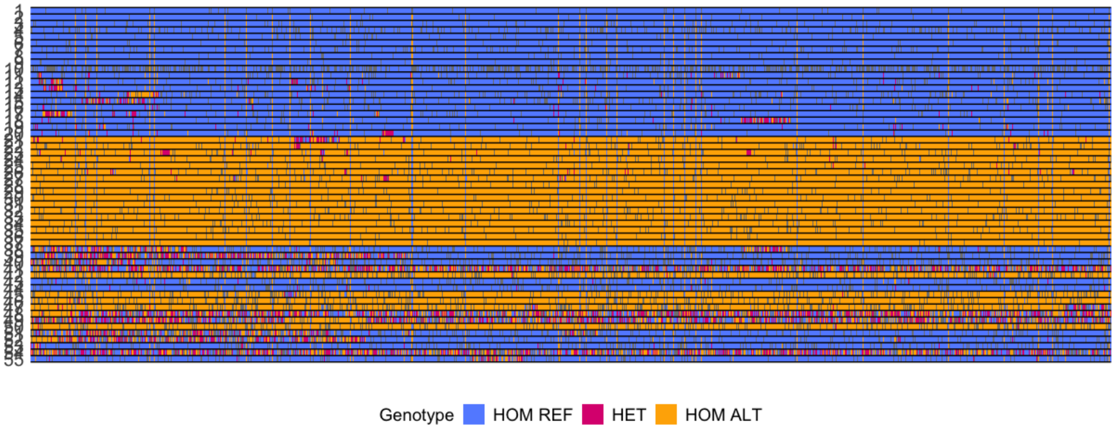

Chromosome Z Section 5 Fixed

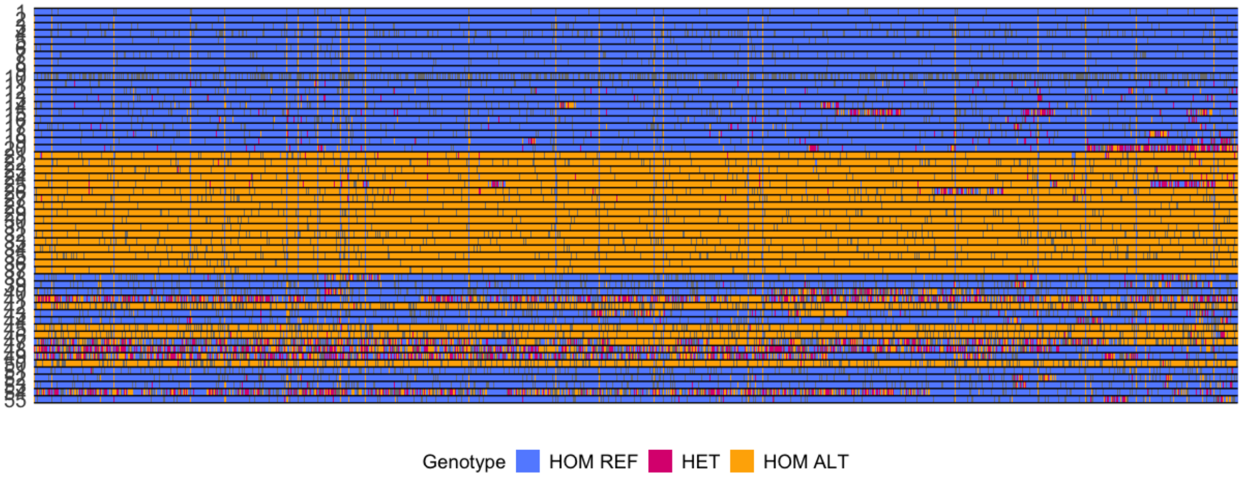

Chromosome Z Section 6 Fixed

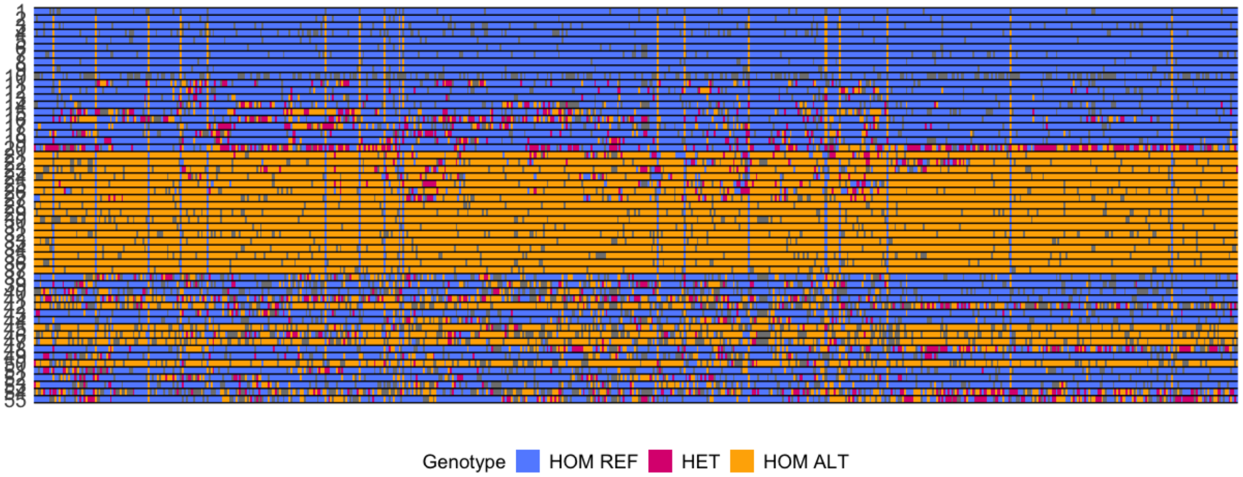

Supplementary Figure 3: Genotypes for every fixed SNP (Fst = 1) across each chromosome plotted in GenotypePlot. Plots have been generated for the 55 resequenced individuals and are presented for every chromosome that had >10 fixed SNPs. Blue represents homozygous

genotypes for the reference allele, yellow is homozygous genotypes for the alternate allele, and pink represents heterozygotes. Each row represents an individual. The top 20 individuals are pure and backcrossed Baltimore Orioles, the next 17 individuals are pure and backcrossed Bullock's orioles, and the last 18 individuals are the putative F1/F2 hybrids. Due to the large number of fixed SNPs on the Z chromosome, plots are broken into 5 sections (approximate 10,000 bp per section).

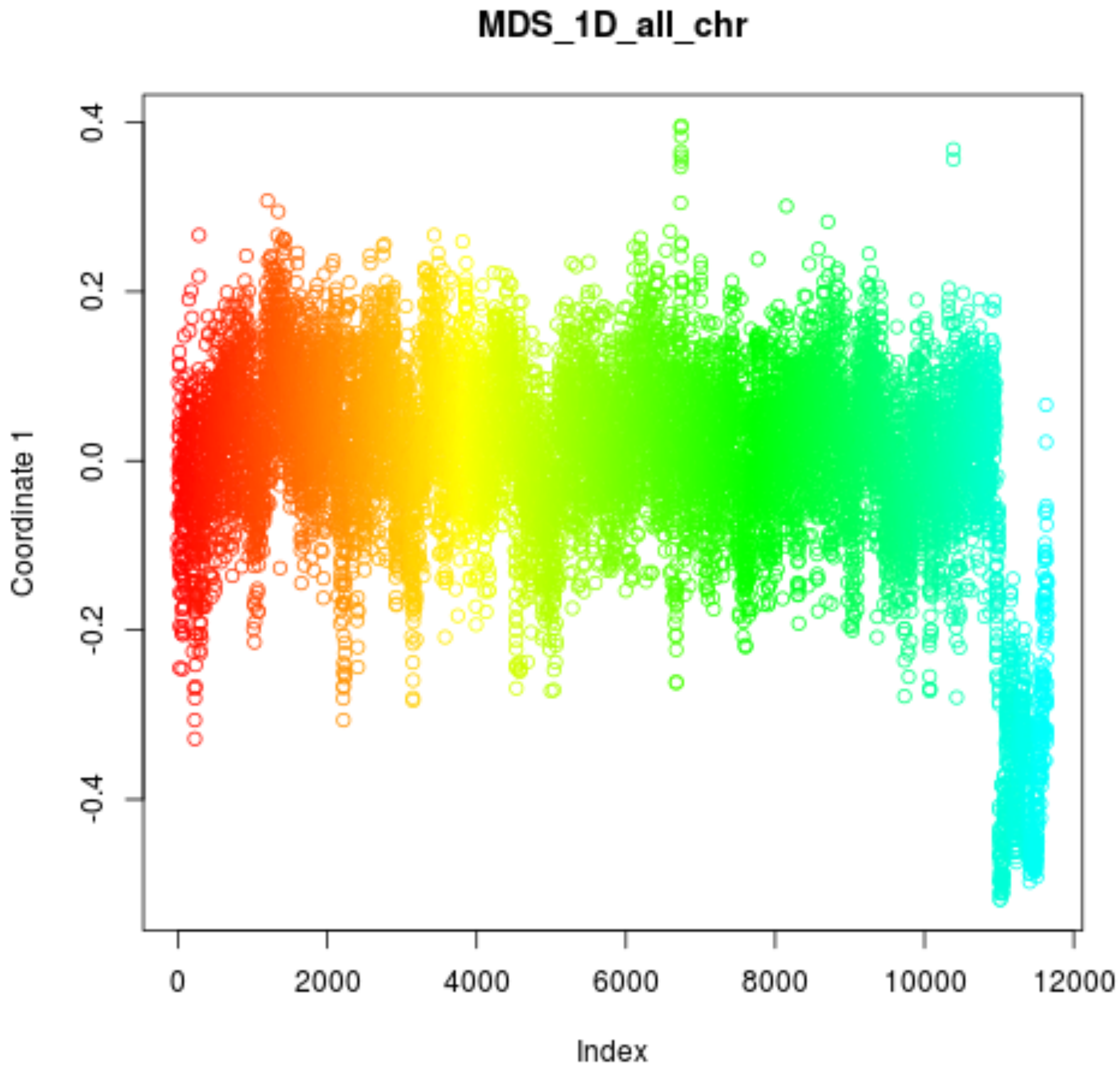

Supplementary Figure 4: Genome-wide plot of MDS estimates. Each point represents a 1000 SNP window. The dip in MDS values (x axis coordinates 10000-12000) correspond with the Z chromosome.

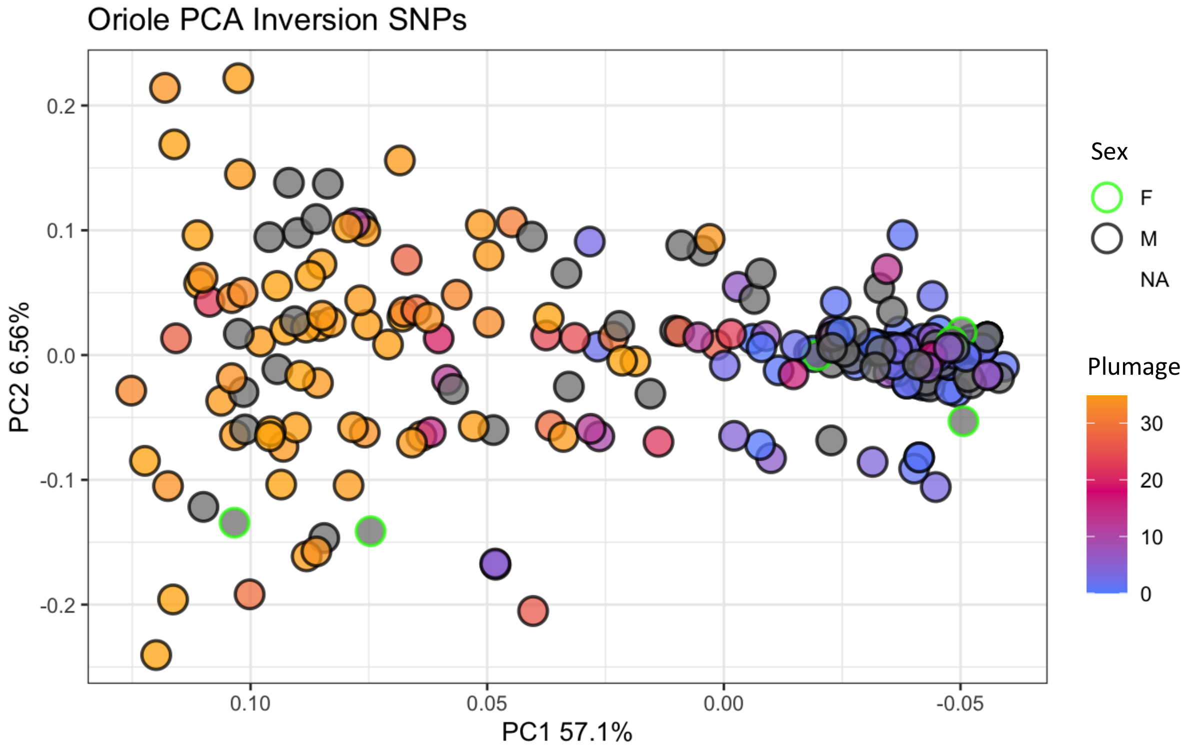

Supplementary Figure 5: PCA of all amplityped individuals based on SNPs putatively linked to the inversion on the Z chromosome. Individuals are color-coded by plumage color (gray points are individuals with no plumage data available). Points circled in green represent females. Although less discrete, there are few individuals falling out in the middle (i.e., heterozygotes for inversion) and none of the individuals in the middle are females.

Supplementary Code:

DATA FILTERING & VARIANT DISCOVERY: Example scripts for the bioinformatics pipeline used for this research can be found at: https://github.com/jenwalshemond/WGSScripts

INVERSION ON THE Z CHROMOSOME: R script for running Lostruct:

```
##Pulling out outliers putatively linked to inversion on Z
devtools::install_github("petrelharp/local_pca/lostruct")
library(lostruct)

snps <- read_tped("Orioles.tped") #only the Z, converted to tped from vcf using VCFTools

#Run PCA in 100 bp windows
snp.pca <- eigen_windows(snps, k=2, win= 100, mc.cores=10)

#Estimate distance matrix for MDS
pcdist <- pc_dist(snp.pca,npc=2, mc.cores=20)
```

```
#Calculate MDS
fit2d <- cmdscale(pcdist, eig=TRUE, k=2)

#Plot MDS1 v MDS2
plot(fit2d$points, xlab="Coordinate 1", ylab="Coordinate 2", col=rainbow(1.2*nrow(pcdist)))

#Plot along chromosome
x <- fit2d$points[,1]
plot(x,ylab="Coordinate 1", main="MDS_1D_all_chr", col=rainbow(2*nrow(pcdist)))

quantile(x, .99) ##0.2563225

outlierMDS<-subset(MDSforZ, MDS>0.2563225)
```

ADMIXTURE MAPPING: See GWAS_GEMMA.sh
(https://github.com/jenwalshemond/WGSScripts)

QUANTIFYING ADMIXTURE PROPORTIONS ACROSS THE GENOME

```
#Index filtered VCF
tabix -p vcf Orioles_filtered_final_093020_55individuals.recode.vcf.gz

#Split VCF by chromosome
bcftools index -s Orioles_filtered_final_093020_55individuals.recode.vcf.gz | cut -f 1 | while read C; do bcftools view -O z -o split.${C}.vcf.gz Orioles_filtered_final_093020_55individuals.recode.vcf.gz "${C}" ; done

##Split each chromosome VCF into individual VCFs containing 100,000 SNPs
##Using program: http://lindenb.github.io/jvarkit/Biostar497922.html
```

Lindenbaum, Pierre (2015): JVarkit: java-based utilities for Bioinformatics. figshare. http://dx.doi.org/10.6084/m9.figshare.1425030

```
##Run admixture on each file and plot across chromosome for individuals
```
