# Supplementary material for: A complex genomic architecture underlies reproductive isolation in a North American oriole hybrid zone
Source: Commun Biol. 2023 Feb 7;6:154. doi: 10.1038/s42003-023-04532-8 (PMC9902562; doi:10.1038/s42003-023-04532-8)
Supplement: Supplementary file 2 — Description of Additional Supplementary Files [file 42003_2023_4532_MOESM2_ESM.pdf]

Description of Additional Supplementary Files

File name: Supplementary Data 1

Description: Sample information for individuals chosen for whole genome sequencing. Sample information includes a voucher ID, a field identification based on plumage score, genotypic class based on previous ddRAD data (see methods), sampling location, total plumage score, the number of raw reads, the percentage of reads mapped to the Myrtle Warbler reference genome, coverage, and percent missing data.

File name: Supplementary Data 2

Description: Sample information for individuals targeted for Amplicon Sequencing. Sample information includes a sample ID, a voucher ID, sex, a field identification based on plumage score, sampling location, and total plumage score.

File name: Supplementary Data 3

Description: Amplicon information for amplotyping of Oriole individuals in this study. Table includes primer name, chromosome location and position of target SNP, amplicon category (melanin-linked, neutral, fixed, inversion linked). For melanin-linked SNPs, we include a column for candidate gene associated with the target SNP.

File name: Supplementary Data 4

Description: Genes associated with putative inversion on the Z chromosome. Table includes gene start and end position, the NCBI protein ID, and the gene name.
