# Supplementary material for: A complex genomic architecture underlies reproductive isolation in a North American oriole hybrid zone
Source: Commun Biol. 2023 Feb 7;6:154. doi: 10.1038/s42003-023-04532-8 (PMC9902562; doi:10.1038/s42003-023-04532-8)
Supplement: Supplementary file 7 — Reporting Summary [file 42003_2023_4532_MOESM7_ESM.pdf]

nature portfolio

Corresponding author(s): Jennifer Walsh

Last updated by author(s): Sep 21, 2022

Reporting Summary

Nature Portfolio wishes to improve the reproducibility of the work that we publish. This form provides structure for consistency and transparency in reporting. For further information on Nature Portfolio policies, see our Editorial Policies and the Editorial Policy Checklist.

Statistics

For all statistical analyses, confirm that the following items are present in the figure legend, table legend, main text, or Methods section.

n/a	Confirmed	
☐	☒	The exact sample size (*n*) for each experimental group/condition, given as a discrete number and unit of measurement
☐	☒	A statement on whether measurements were taken from distinct samples or whether the same sample was measured repeatedly
☒	☐	The statistical test(s) used AND whether they are one- or two-sided<br>*Only common tests should be described solely by name; describe more complex techniques in the Methods section.*
☒	☐	A description of all covariates tested
☒	☐	A description of any assumptions or corrections, such as tests of normality and adjustment for multiple comparisons
☐	☒	A full description of the statistical parameters including central tendency (e.g. means) or other basic estimates (e.g. regression coefficient) AND variation (e.g. standard deviation) or associated estimates of uncertainty (e.g. confidence intervals)
☒	☐	For null hypothesis testing, the test statistic (e.g. *F*, *t*, *r*) with confidence intervals, effect sizes, degrees of freedom and *P* value noted<br>*Give P values as exact values whenever suitable.*
☒	☐	For Bayesian analysis, information on the choice of priors and Markov chain Monte Carlo settings
☒	☐	For hierarchical and complex designs, identification of the appropriate level for tests and full reporting of outcomes
☒	☐	Estimates of effect sizes (e.g. Cohen's *d*, Pearson's *r*), indicating how they were calculated

Our web collection on statistics for biologists contains articles on many of the points above.

Software and code

Policy information about availability of computer code

Data collection	No software used for data collection
Data analysis	Several programs were used for data analysis. These programs are described in detail and cited in the manuscript. We provide a list below. All relevant code is included in the supporting document. We will additionally include a link to GitHub containing bioinformatic scripts used in this study.<br><br>Data Filtering/Variant Discovery: FastQC, AdapterRemoval V2.1.1, CUTADAPT V 2.1, FASTX Toolkit, BWA, QUALIMAP, SAMTOOLS, PICARD TOOLS, GATK, BCFTOOLS<br><br>Summary Statistics: R, VCFTools<br><br>Inversion Analyses: R, LUMPY, PANTHER<br><br>Admixture Mapping: GEMMA, BEAGLE, R<br><br>Gene ID: Geneious, R, Uniprot<br><br>Introgression patterns: ADMIXTURE, HZAR

For manuscripts utilizing custom algorithms or software that are central to the research but not yet described in published literature, software must be made available to editors and reviewers. We strongly encourage code deposition in a community repository (e.g. GitHub). See the Nature Portfolio guidelines for submitting code & software for further information.

Data

Policy information about availability of data

All manuscripts must include a data availability statement. This statement should provide the following information, where applicable:

- Accession codes, unique identifiers, or web links for publicly available datasets
- A description of any restrictions on data availability
- For clinical datasets or third party data, please ensure that the statement adheres to our policy

Relevant scripts are included in the supporting document. Basic bioinformatic pipelines are deposited in a GitHub repository (link in supporting info). Filtered VCFs will be deposited on Dryad. Raw sequences will be provided upon request.

Human research participants

Policy information about studies involving human research participants and Sex and Gender in Research.

Reporting on sex and gender	NA
Population characteristics	NA
Recruitment	NA
Ethics oversight	NA

Note that full information on the approval of the study protocol must also be provided in the manuscript.

Field-specific reporting

Please select the one below that is the best fit for your research. If you are not sure, read the appropriate sections before making your selection.

☐ Life sciences ☐ Behavioural & social sciences ☒ Ecological, evolutionary & environmental sciences

For a reference copy of the document with all sections, see nature.com/documents/nr-reporting-summary-flat.pdf

Ecological, evolutionary & environmental sciences study design

All studies must disclose on these points even when the disclosure is negative.

Study description	Whole genome and amplicon sequencing of Baltimore and Bullock's orioles across a hybrid zone to characterize the genomic architecture of reproductive isolation.
Research sample	Icterus galbula and Icterus bullocki tissue samples. Whole genome sequencing included 60 males (which ensures equal coverage of the sex chromosomes and autosomes). Amplicon sequencing is of both male and female individuals sampled across the hybrid zone
Sampling strategy	Samples were collected as part of a re-sampling effort of the Platte River transect, which runs across the oriole hybrid zone and was originally sampled in the 1950's. This sampling endeavor was geared toward studying the temporal stability of the zone. Birds were collected predominantly using playback and all tissue samples and associated study skins are stored at the Cornell University Museum of Vertebrates
Data collection	Phenotypes for collected specimens were scored by VR (see methods). Tissues and study skins from the collected individuals are archived at the Cornell University Museum of Vertebrates. DNA was extracted by JW and BB using standard protocols (detailed in methods). Sequencing was done at Cornell
Timing and spatial scale	Samples were collected in the summer (breeding season) of 2016-2018, to ensure that birds sampled were resident. Sites were chosen to replicate geographic sampling from a 1950's time point.
Data exclusions	For whole genome sequencing: four individuals were removed due to low coverage and data quality (high missing data) and one individual was removed due to high relatedness to another male. For amplicon sequencing: we filtered to exclude all individuals with a mean depth of less than 2, which removed all negative controls and 8 individuals. Finally, sites with more than 50% missing data were removed and 94 sites (78%) were retained.
Reproducibility	All scripts and raw data can be made available to allow for reproducibility of results. We have not re-run the pipelines, due to lengthy and expensive computational time, but the bioinformatic pipelines are robust and have been used extensively. Most findings are supported by multiple, complimentary analyses (i.e., the inversion).
Randomization	NA

Blinding	Blinding, in the traditional experimental design sense, was not possible during specimen acquisition because we specifically targeted males of two species (Baltimore and Bullock's Orioles) and their hybrids across a transect of their hybrid zone. In areas where both species co-occured, we did not preferentially collect one species over the other, or preferentially collect hybrids over parentals; we attempted to collect all target birds that were within range. The ecology of these species further reduced potential bias during specimen collection. Orioles typically occupy the canopy of tall trees, making it difficult to distinguish subtle plumage characters in the field. This further reduced the possibility of targeting hybrids over parentals or one species over the other in areas of sympatry.

Did the study involve field work? ☒ Yes ☐ No

Field work, collection and transport

Field conditions	Field work spanned a roughly 700km east-west transect along the Platte and South Platte Rivers from Blair, Nebraska, USA (in the east) to Greeley, Colorado, USA in the west. Along this transect, habitat transitions from relatively continuous eastern deciduous forest in the east, to arid grasslands and farmlands with a thin band of riparian forest concentrated along the Platte and South Platte Rivers in the west. Elevation at the eastern edge of the transect is ~300m and reaches ~1450m at the western edge. All field work was conducted during the breeding season (late May-June).
Location	Field sites were spaced roughly 80km apart along the entire length of the transect. From east to west, locations include: Blair (N 41 29' 27.87" W 96 3' 56.21"), Schuyler (N 41 24' 21.28" W 97 4' 59.06"), Silver Creek (N 41 19' 56.53" W 97 35' 58.64"), Grand Island (N 40 48' 51.55" W 98 25' 35.98"), Elm Creek (N 40 41' 5.83" W 99 23' 27.67"), Gothenburg (N 40 56' 57.70" W 100 16' 7.06"), Sutherland (N 41 8' 58.53" W 101 4' 26.54"), Big Springs (N 41 4' 22.74" W 101 59' 9.10"), Crook (N 40 50' 20.01" W 102 48' 43.35"), Fort Morgan (N 40 16' 57.07" W 103 42' 9.09"), Greeley (N 40 22' 2.14" W 104 26' 28.48").
Access & import/export	Field work at all sites was approved by local landowners, by Cornell University's IACUC, and by the US Fish and Wildlife Service at both state (Nebraska permit numbers: 579, 1025, 1091; Colorado permit numbers: 17TRb2328, 18TRb2328), and Federal levels (Federal permit number: MB020189). All specimens collected are currently housed at the Cornell University Museum of Vertebrates.
Disturbance	Bullock's and Baltimore Orioles are two common and abundant species along the Platte and South Platte Rivers. We minimized our impact to local breeding populations by collecting males (females bare the bulk of parental care responsibilities), working in the early part of the breeding season (prior to peak demands of nestlings), and by collecting, at most, 10 males of each species at a locality per year. Collectors acquiring samples also had 25+ years of experience collecting and processing birds in the field, further minimizing the loss of individual birds as a result of inadequate field experience.

Reporting for specific materials, systems and methods

We require information from authors about some types of materials, experimental systems and methods used in many studies. Here, indicate whether each material, system or method listed is relevant to your study. If you are not sure if a list item applies to your research, read the appropriate section before selecting a response.

Materials & experimental systems

n/a	Involved in the study	
☒	☐	Antibodies
☒	☐	Eukaryotic cell lines
☒	☐	Palaeontology and archaeology
☐	☒	Animals and other organisms
☒	☐	Clinical data
☒	☐	Dual use research of concern

Methods

n/a	Involved in the study	
☒	☐	ChIP-seq
☒	☐	Flow cytometry
☒	☐	MRI-based neuroimaging

Animals and other research organisms

Policy information about studies involving animals; ARRIVE guidelines recommended for reporting animal research, and Sex and Gender in Research

Laboratory animals	NA
Wild animals	All birds used in this study were collected using a shotgun, and are currently housed at the Cornell University Museum of Vertebrates. Collected birds represent a scientific standard for natural history collections and serve as vouchers for this and other studies that rely on plumage, genetics, isotopes, parasites, etc. We targeted male Baltimore and Bullock's Orioles and their hybrids. All birds were reproductively mature and in their first or later breeding season. Orioles were either prepared in the field with tissues preserved in liquid nitrogen, or flash frozen on dry ice and prepared in the lab after the field season.
Reporting on sex	All orioles used in this study were male.

Field-collected samples	No laboratory work for this study relied on housing or maintaining wild-caught living animals.
Ethics oversight	Cornell University's Institutional Animal Care and Use Committee (IACUC), state-level US Fish and Wildlife approval in Nebraska and Colorado (Nebraska permit numbers: 579, 1025, 1091; Colorado permit numbers: 17TRb2328, 18TRb2328), and federal-level approval from US Fish and Wildlife Service (federal migratory bird permit number: MB020189).

Note that full information on the approval of the study protocol must also be provided in the manuscript.
